# Areca palm yellow leaf disease (YLD) detection and severity grading from UAV Imagery with an enhanced YOLOv10s model

**DOI:** 10.3389/fpls.2026.1899720

**Published:** 2026-09-24

**Authors:** Zhengfa Yang, Mingxin Ren, Yuhao Hu, Shuxin Zhu, Huanliang Xu, Zhaoyu Zhai, Qinghua Tang, Hongfeng Yu

**Affiliations:** 1 Sanya Institute of Nanjing Agricultural University, Sanya, China; 2 College of Smart Agriculture (College of Artificial Intelligence), Nanjing Agricultural University, Nanjing, China; 3 Chinese Academy of Tropical Agricultural Sciences, Wenchang, China; 4 College of Engineering, Nanjing Agricultural University, Nanjing, China

**Keywords:** areca palm, disease severity grading, UAV imagery, yellow leaf disease, YOLOv10s

## Abstract

Efficient and accurate assessment of the severity of areca palm (*Areca catechu L.*) yellow leaf disease (YLD) is essential for early prevention, precision pesticide application, and the sector’s long-term viability. However, accurate detection of YLD from UAV imagery remains a significant challenge owing to severe canopy occlusion, subtle early-stage lesions, intricate background clutter, and minimal inter-class variance. Given these challenges, we introduce an optimized YOLOv10s model for YLD detection. First, a non-parametric SimAM attention mechanism is incorporated into the backbone to engineer the C2f_SimAM module; this design optimizes feature extraction within lesion areas while successfully mitigating intricate background noise. Second, the Histogram Transformer Block (HTB) is embedded into the deep feature extraction stage, which strengthens multi-scale feature extraction via dynamic-range histogram self-attention, thereby advancing the network’s capability in capturing diminutive targets. Finally, we incorporate a Soft-NMS-GIoU strategy to alleviate localization bias and reduce missed detections under dense occlusion. The upgraded YOLOv10s outperforms the baseline by 2.4 percentage points, reaching 92.9% mAP0.5. Precision and recall reach 88.7% and 86.8%, respectively, at a frame rate of 88.350 FPS. The confusion between healthy and mild YLD categories is significantly reduced, with their respective recall rates improved by 3% and 7%. Field deployment in areca palm plantations demonstrates the model’s proficiency in pinpointing disease hotspots and generating visualization maps, confirming its practicality and reliability. The methodology developed in this work offers a robust technical foundation for the large-scale automated monitoring and precise management of YLD.

## 1 Introduction

As a significant tropical economic crop in China, the areca palm (*Areca catech*u L.) is predominantly cultivated across Hainan, Guangxi, and Taiwan Provinces, with Hainan constituting the primary production hub. According to the *Hainan Statistical Yearbook 2024* (Hainan Provincial Bureau of Statistics, 2024), the cultivation area of areca palm in Hainan exceeded approximately 266,667 ha, constituting almost a third of the entire tropical crop zone across the province. The region’s annual areca nut output has escalated to millions of tons. However, with the expansion of large-scale cultivation, disease issues have become increasingly prominent. Areca palm yellow leaf disease (YLD) severely jeopardizes the long-term sustainability of the areca sector. According to a field investigation conducted by the Coconut Research Institute of the Chinese Academy of Tropical Agricultural Sciences (CATAS) (Coconut Research Institute, Chinese Academy of Tropical Agricultural Sciences, 2024), the affected area of YLD in Hainan Province exceeded approximately 46,667 ha in 2024, resulting in substantial yield losses and significant economic impact. YLD is a highly destructive disease characterized by rapid spread and severe damage. It typically manifests as progressive leaf chlorosis and inhibited plant growth, and in severe cases may lead to complete plant death, ultimately resulting in substantial yield losses or even total crop failure at the plantation scale (Tang et al., 2022a). Therefore, early detection of YLD is pivotal to optimizing disease management and safeguarding stable production.

Traditional monitoring methods primarily rely on manual field inspection, where technicians assess plant health through visual observation. However, due to their inherent high labor demands and prolonged operational periods, these approaches present significant challenges for extensive regional deployment. In addition, manual identification depends heavily on expert experience and is inherently subjective, resulting in limited consistency and reliability.

With the rapid development of digitalization technologies, UAV-based remote sensing now plays a pivotal role in agricultural monitoring, owing primarily to its inherent flexibility and broad swath width (Li et al., 2026; Zeng et al., 2026). By equipping UAVs with high-resolution sensors and integrating computer vision techniques, automated disease detection has become a major research focus in smart agriculture. Historically, crop disease detection relied mainly on traditional machine learning frameworks. These methods required users to manually extract visual features—such as color, texture, and shape—before performing classification with algorithms like support vector machines (SVM) and random forests (RF) (Tan et al., 2025; Wolffang et al., 2026). However, crop images captured under natural conditions often exhibit significant variations in illumination, complex backgrounds, and subtle inter-class differences. These factors heavily compromise the performance of traditional methods. As a result, these approaches are limited in capturing discriminative features and often exhibit poor generalization in real-world scenarios.

The continuous evolution of deep learning has transformed plant disease detection, leading to the widespread adoption of convolutional neural networks (CNNs). Through multi-layer convolutional operations, CNN-based models autonomously extract feature hierarchies, thereby reducing reliance on handcrafted features and improving detection accuracy. Building upon this foundation, object detection algorithms have been widely adopted in agricultural applications, including Faster R-CNN, SSD, and the YOLO family (Ariza-Sentís et al., 2024; Bhandari and Etienne, 2026). Among these, the two-stage Faster R-CNN framework delivers high detection accuracy at the expense of inference speed, thereby restricting its capability in extensive, real-time agricultural scenarios (Naufaldihanif et al., 2025). In contrast, the YOLO family adopts a one-stage detection paradigm that enables end-to-end optimization of localization and classification, achieving a favorable trade-off between speed and accuracy. Consequently, YOLO models have been extensively applied in agricultural object detection.

Driven by the escalating need for prompt and high-resolution perception in precision agriculture, merging UAV remote sensing with deep learning algorithms has emerged as a vibrant research frontier for crop disease detection. Recent studies have demonstrated the feasibility of merging UAV imagery with advanced detection models. For instance, Villalobos-Salazar et al. (2026) utilized UAV remote sensing and the YOLOv8 model for early and automated pest monitoring, achieving an accuracy of 94.4% on a self-constructed dataset. Liu et al. (2025) integrated ultra-low-altitude UAV phenotyping with deep learning to accurately detect wheat Fusarium head blight, achieving an average precision (AP) of 92.6% for infected spikes. Dang et al. (2025) developed a lightweight convolutional network tailored for maize leaf disease identification via UAV imagery. By incorporating depthwise separable convolutions, coordinate attention mechanisms, and channel pruning into the YOLOv10n framework, the model reaches 94.1% mean average precision (mAP0.5) with only 2.99 million parameters. Similarly, to target occluded and tiny litchi pests, Liang et al. (2025) developed a lightweight YOLOv7-MSRSF model integrated with ultra-low-altitude UAV remote sensing. Through frequency-domain feature fusion and detection head optimization, their approach reaches 96.1% mAP0.5. These studies collectively highlight that combining deep learning with UAV remote sensing is both broadly applicable and highly effective in agricultural monitoring. Beyond overall detector optimization, recent studies have increasingly explored targeted architectural modifications, including attention mechanisms, loss redesign, and feature fusion strategies, to alleviate specific limitations such as weak feature representation, scale variation, and localization uncertainty in agricultural scenarios. In 2025, Trinh et al. (2024) proposed an Alpha-EIoU loss to refine bounding box regression in rice disease detection. Feng et al. (2025) integrated DSConv, BiFPN, and CBAM attention with a Focal-EIoU loss to enable lightweight cotton disease identification. Chen et al. (2025) combined Gold-YOLO feature aggregation with EMA attention and GIoU loss for weed detection in rice paddies. Zhao et al. (2025) developed a multi-scale spatial attention module (MCSA) coupled with WIoUv3 loss to enhance multi-crop disease detection in diverse field settings. These methodological advances—spanning loss function design, attention mechanisms, and multi-scale feature fusion—have yielded measurable performance gains, yet their applicability to orchard-scale, tree-structured crops with distinct disease phenotypes remains largely untested.

A closer examination reveals, however, that these SOTA methods have been predominantly validated on row crops, leaving UAV-based YLD detection in areca palm plantations—with its distinct occlusion, lesion subtlety, and class-ambiguity challenges—largely unexplored. Compared with staple crops, areca palm presents unique challenges for disease detection in UAV imagery due to its complex canopy structure, uneven planting density, and diverse manifestations of yellowing symptoms, which are often confounded with illumination variation and shadow effects. These factors significantly affect the accurate assessment of disease severity. Moreover, areca palm targets in UAV images typically exhibit characteristics such as small object size, multi-scale distribution, and strong background interference, further increasing detection difficulty. The distinction between different disease severity levels mainly relies on subtle variations in leaf coloration and localized texture patterns. However, such fine-grained features are difficult to capture effectively under complex field conditions. In addition, healthy areca palms may exhibit natural yellowing of older leaves, which visually resembles early-stage disease symptoms, thereby bringing considerable ambiguity in classification. These subtle inter-class differences are easily overlooked or misinterpreted. Therefore, enabling the model’s precise focus on disease-relevant regions and boosting its fine-grained feature extraction capacity are critical for improving YLD detection accuracy.

Aimed at overcoming the difficulties of miniature object detection, weak feature representation, intricate background clutter, and limited capability in distinguishing disease severity levels in UAV imagery, this study proposes an improved YOLOv10-based approach for YLD detection. Rather than simply aggregating established techniques, each structural modification is explicitly driven by physical imaging constraints in precision plant protection. The primary contributions of this work are summarized as follows:

- A parameter-free SimAM attention is embedded into the C2f module of the YOLOv10 backbone (C2f_SimAM), directing feature learning toward lesion areas and suppressing background interference without adding parameters.
- A Histogram Transformer Block (HTB) with dynamic-range self-attention is introduced in the deep feature extraction stage to strengthen multi-scale lesion representations, particularly benefiting small-target detection.
- To reduce localization bias and missed detections under dense occlusion, CIoU loss is replaced with GIoU loss, and a Soft-NMS-GIoU strategy is adopted for prediction box filtering.
- Extensive experiments on a UAV-captured areca palm dataset yield 0.929 mAP0.5 and 0.887 precision, outperforming existing detectors. Real-world plantation deployment further demonstrates the model’s ability to accurately locate disease outbreak centers, offering effective support for precision disease management.

## 2 Materials and methods

### 2.1 Study site and data acquisition

Field data were collected at an areca palm plantation of the Wenchang-based Coconut Research Institute, Chinese Academy of Tropical Agricultural Sciences (CATAS), in Hainan Province, China (110°46′E, 19°33′N), during July 23–24, 2024. A DJI Mavic 3 Pro UAV equipped with an onboard 20-megapixel camera was used to capture aerial video streams, from which individual image frames were extracted at a native resolution of 3,840 × 2,160 pixels. To ensure comprehensive documentation of the areca palm canopy, the camera maintained a strict nadir viewing angle (90°). Flight missions were executed at a constant speed of 2 m/s at an altitude of 10 m above the canopy. All aerial surveys were conducted between 10:00 and 14:00 under clear-sky conditions to ensure stable, uniform solar illumination, thereby minimizing shadow interference and color distortion across the captured dataset.

### 2.2 Dataset construction

After excluding blurred images and those with significant scale variations, a total of 2,005 high-quality samples were retained. These images were labeled manually using the LabelImg software (**Figure 1A**), and the resulting dataset was exported in a YOLO-compliant format.

The severity grading criteria were established with reference to the official *Technical Information Sheet for Prevention and Control of Areca Palm Yellow Leaf Disease* (hereinafter referred to as the Information Sheet) (Tang et al., 2022b) issued by the Hainan Provincial Major Science and Technology Project Team for Areca Palm Pests and Diseases, with technical support from the Coconut Research Institute of CATAS, Hainan University, and the Institute of Plant Protection of Hainan Academy of Agricultural Sciences. According to this Information Sheet, the disease is classified into four operational grades based on the proportion of yellowed canopy area (*P*): Grade 1 (*P* < 20%), Grade 2 (20% ≤ *P* < 50%), Grade 3 (50% ≤ *P* < 80%), and Grade 4 (*P* ≥ 80%), with Grades 3 and 4 additionally characterized by bunchy top symptoms and reduced fruiting capacity.

In this study, to accommodate the fine-grained detection requirements of UAV-based remote sensing and to better capture subtle early-stage chlorotic lesions, we adopted a refined three-level classification scheme following the Information Sheet:

1. Healthy (*P* < 1%): The canopy leaves exhibit a normal deep green color with no visible chlorosis or lesions, and the crown remains intact and lush (**Figure 1B**).
2. Mild YLD (1% ≤ *P<* 10%): Sporadic yellowing appears on the middle and lower leaves, with the symptomatic area accounting for less than 10% of the total canopy. The heart leaves remain largely unaffected (**Figure 1C**).
3. Severe YLD (*P* ≥ 10%): The yellowed area exceeds 10% of the canopy, or bunchy top symptoms occur. Such plants exhibit significant growth decline and a marked reduction in production capacity (**Figure 1D**).

**Figure 1:**
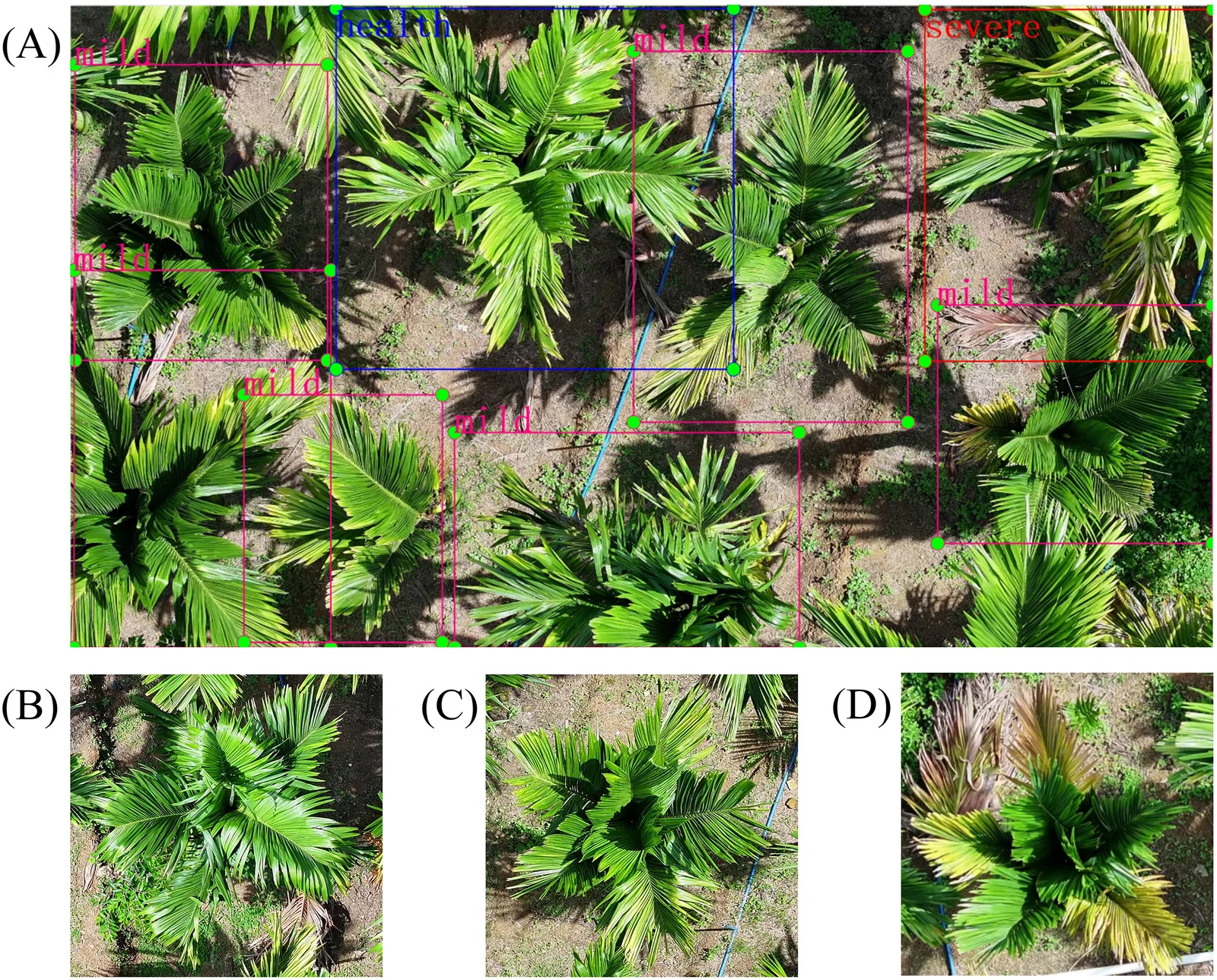
Data collection and classification samples: **(A)** manual annotation, **(B)** healthy plant, **(C)** mild YLD, and **(D)** severe YLD.

It should be noted that our three-level scheme corresponds to the official Information Sheet as follows: Healthy corresponds to asymptomatic plants; Mild roughly aligns with the lower end of Grade 1 (1%–10%); and Severe encompasses the upper portion of Grade 1 (≥10%) through Grades 2–4, focusing on the distinction between asymptomatic/mild cases and advanced disease stages, which is most critical for early warning and precision management decisions.

The entire image set was randomly allocated into training (70%), validation (15%), and testing (15%) subsets, comprising 1,403, 301, and 301 images, respectively. The detailed distribution of category-specific samples is summarized in **Table 1**. The number of annotation boxes for Healthy, Mild YLD, and Severe YLD categories in the training set are 6,082, 4,296, and 3,938, respectively. The class distributions in the testing and validation sets remain consistent with those in the training set. Overall, the sample sizes across the three categories are relatively balanced, demonstrating that the dataset provides sufficient representative samples to meet the requirements for robust model training and evaluation.

**Table 1:** Statistical summary of label annotations in the areca palm YLD detection dataset.

Class	Training set	Test set	Validation set
Health	6,082	1,149	1,375
Mild YLD	4,296	908	894
Severe YLD	3,938	899	818

Furthermore, a comprehensive statistical analysis of all annotation boxes in the dataset was conducted, as illustrated in **Figure 2**. Specifically, the bar chart in **Figure 2A** shows the number of instances per category. The overlaid bounding box distribution in **Figure 2B** provides a visual depiction of target scale variability. The spatial alignment of bounding box centers (x,y) within normalized image coordinates is presented in **Figure 2C**, whereas the covarying patterns between box dimensions (width and height) are analyzed in **Figure 2D**. It can be observed that the centers of the bounding boxes uniformly cover the entire normalized coordinate space [0,1] without significant positional bias. This wide spatial distribution effectively fortifies the model against spatial variations across images. Furthermore, the box dimensions scale proportionally, exhibiting extensive variations in absolute magnitude. This multi-scale nature effectively empowers the network to achieve superior localization accuracy and broader domain adaptability.

**Figure 2:**
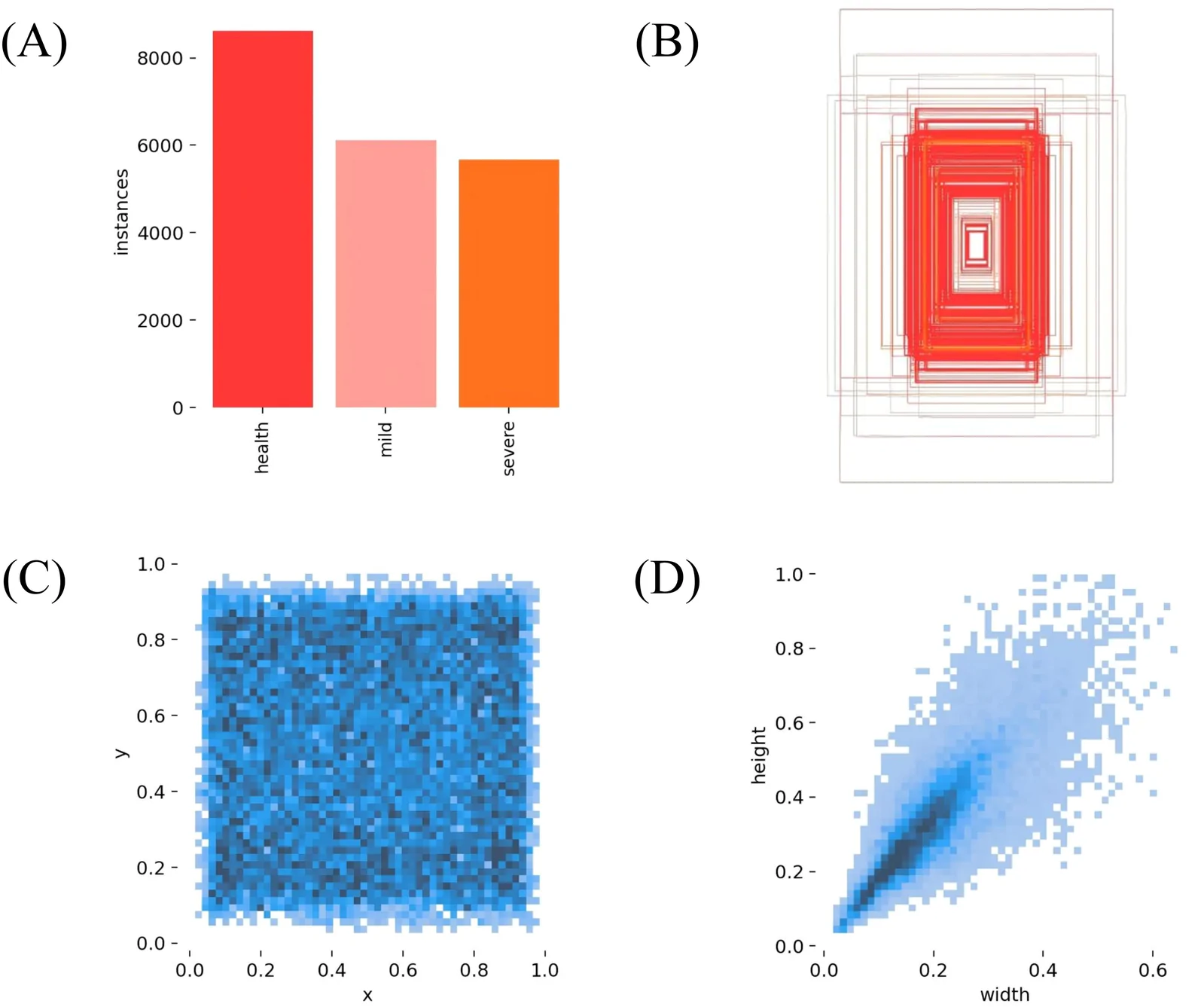
A statistics summary of the labelled bounding boxes: **(A)** number of instances per category, **(B)** bounding box distribution, **(C)** spatial distribution of bounding box centers within images, **(D)** aspect ratio distribution of target regions.

The dataset presents several significant challenges for areca palm detection. First, we observe inter-plant occlusion (see **Figure 3A**). Dense planting leads to extensive canopy overlap, making it difficult to distinguish and delineate individual tree crowns. Second, we also meet the small object detection issues (see **Figure 3B**). Due to the flight altitude and varying growth stages, some areca palm canopies contain limited pixel information, resulting in the loss of fine-grained texture and structural details. Third, visual features of target plants can be easily confounded by environmental noise, such as field weeds, bare soil, and other non-target vegetation (see **Figure 3C**).

**Figure 3:**
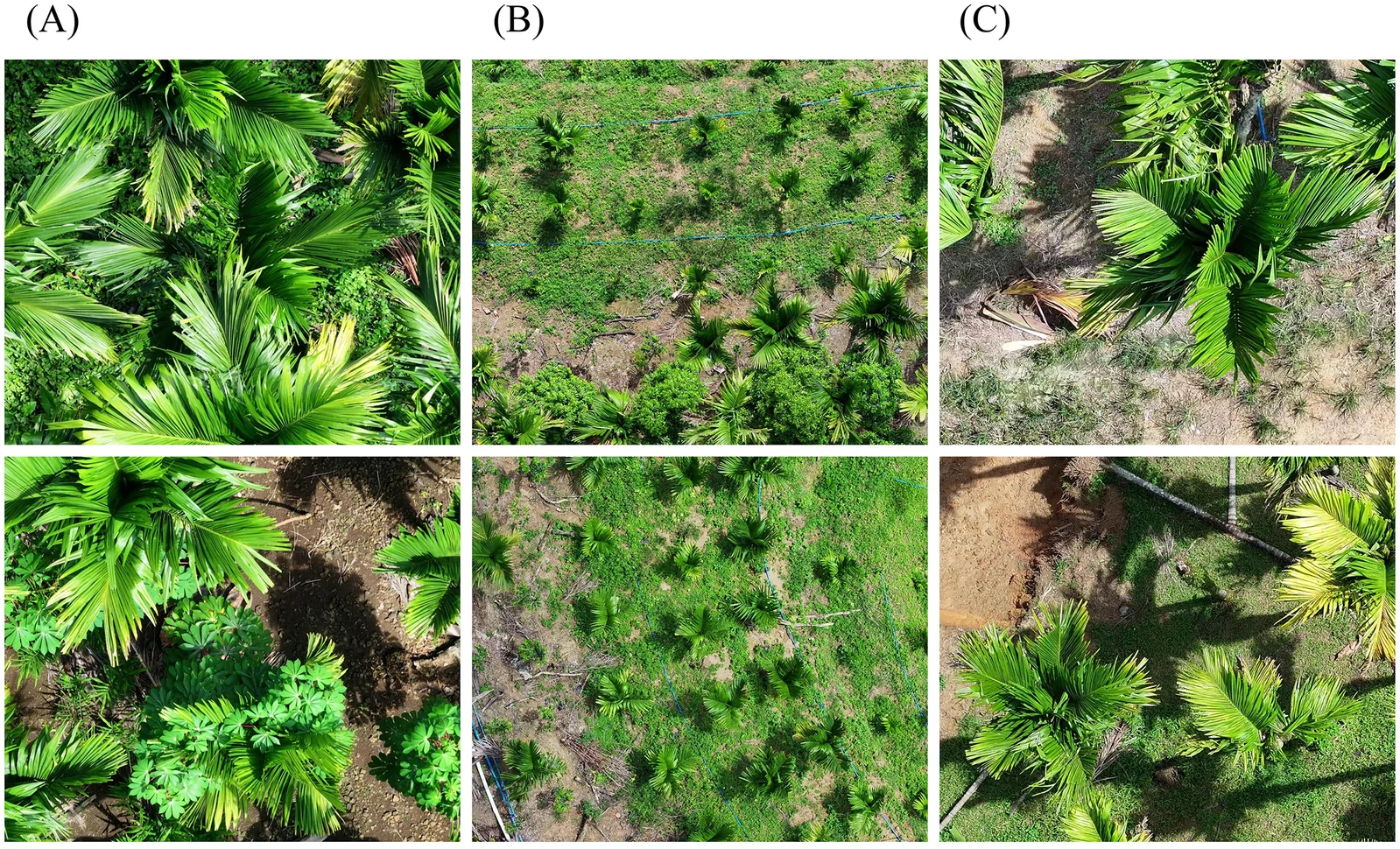
Visualization of hard examples: **(A)** occlusion, **(B)** small targets, **(C)** complex background.

During training, all augmentations were applied online using the Ultralytics YOLO framework. The pipeline included Mosaic augmentation (applied with probability 1.0, disabled for the final 10 epochs via close_mosaic=10), HSV color jittering (hue ±0.015, saturation ±0.7, value ±0.4), random scaling and translation (± 0.5 and ±0.1, respectively), and random horizontal flipping (probability 0.5). MixUp, rotation, shear, perspective, and vertical flipping were not employed. No test-time augmentation was used to ensure inference efficiency.

### 2.3 Model selection and improvement

#### 2.3.1 YOLOv10 baseline model

YOLOv10, proposed by Wang et al. (2024), is organized into three functional modules—Backbone, Neck, and Head—to enable real-time, end-to-end object detection. Building upon the YOLOv8 (Varghese and M., 2024) framework, YOLOv10 integrates a uniform dual-assignment approach, eliminating unnecessary computational overhead. Aimed at minimizing computational redundancy, YOLOv10 implements a Spatial-Channel decoupled Downsampling (SCDown) mechanism alongside the Compact Inverted Block (CIB), both of which serve to strengthen feature representation. To broaden the receptive field and optimize global information acquisition while maintaining a lightweight architecture, large-kernel convolutions and Partial Self-Attention (PSA) modules are utilized. YOLOv10 offers six scaled versions: n, s, m, b, l, and x. Considering the accuracy and computational efficiency trade-off, this study selects YOLOv10s as the baseline model for further improvement.

#### 2.3.2 Proposed enhancements for the YOLOv10 framework

This study optimizes the YOLOv10s architecture in three structural directions for YLD detection. First, a parameter-free SimAM attention module is embedded into the C2f structure, utilizing maximum-value pooling to calculate and refine 3-D spatial-channel feature correlations. Second, the backbone’s deep layers incorporate a C2fCIB structure fused with a Histogram Transformer Block (HTB). This mechanism leverages dynamic range histogram self-attention to map feature pixels into distinct intensity-based bins, thereby enforcing an explicit segregation between foreground lesion zones and complex environmental backgrounds (e.g., soil, weeds, and shadows) (Sheng et al., 2025). Subsequently, cross-bin global attention interaction aggregates the multi-scale features of miniature lesions. Finally, during bounding box regression and filtering, the GIoU loss function—which utilizes the smallest enclosing box as a geometric penalty to provide continuous gradients—and Soft-NMS replace the conventional NMS. **Figure 4** delineates the enhanced YOLOv10s framework.

**Figure 4:**
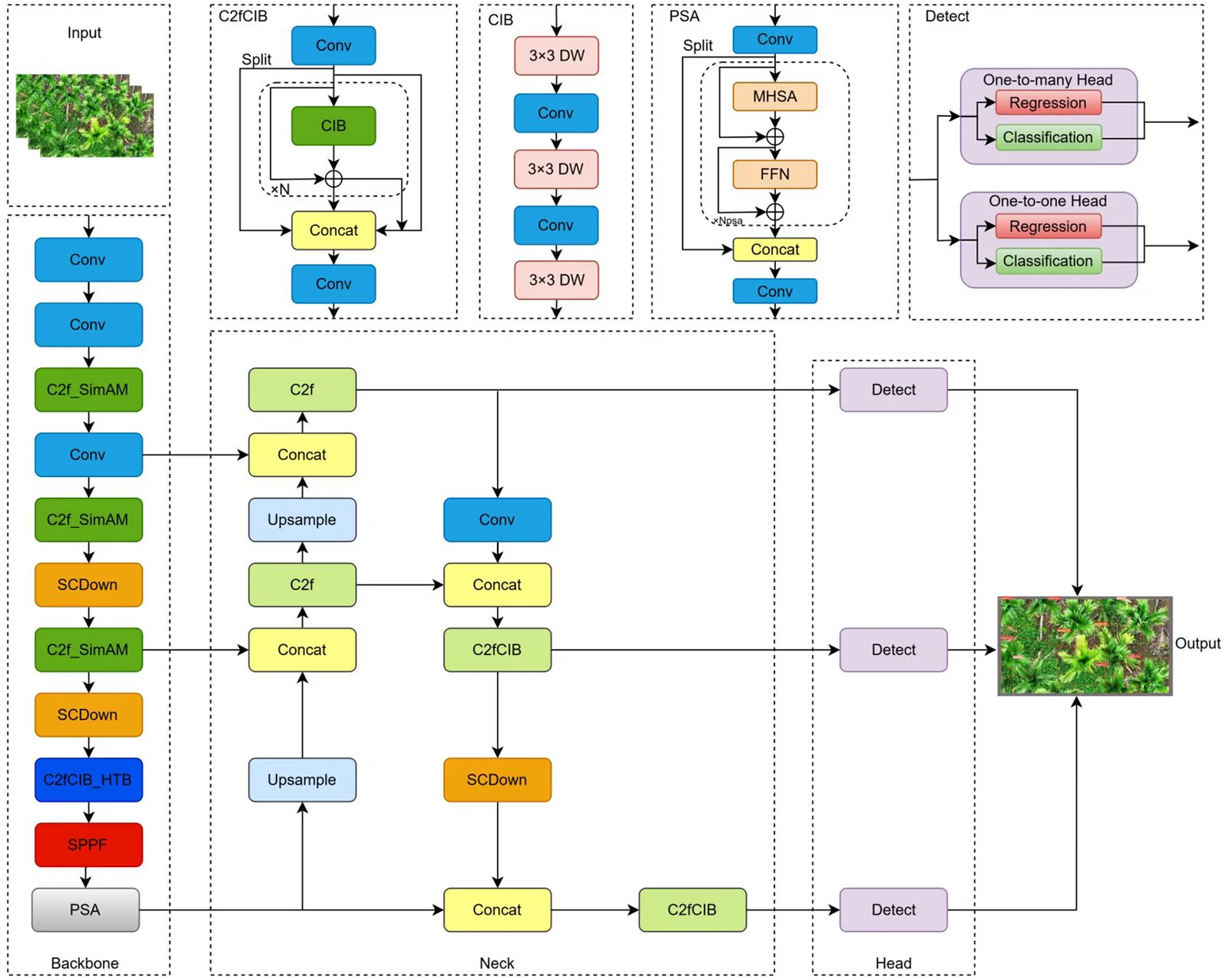
The architectural design of the proposed model.

##### 2.3.2.1 The C2f_SimAM module

The early pathological features of YLD are subtle and easily obscured by complex backgrounds, such as weeds and soil. Furthermore, areca palms are typically planted in high density, resulting in severe mutual shading of leaves. Traditional attention mechanisms, such as Squeeze-and-Excitation (SE) (Hu et al., 2018) and Convolutional Block Attention Module (CBAM) (Woo et al., 2018), primarily perform feature optimization along a single dimension (either channel or spatial), making it challenging to achieve precise capture of critical features. To overcome this limitation, this study proposes the C2f_SimAM module by integrating the Simple, Parameter-Free Attention Module (SimAM) (Yang et al., 2021; Yao et al., 2025) into the original C2f structure, with its detailed architecture illustrated in **Figure 5A**. In this configuration, the SimAM mechanism, appended to the terminal output of the baseline C2f structure, processes the data flow. This occurs immediately after the incoming feature maps undergo a channel-splitting routine driven by a 1×1 convolutional layer and a *Split* operation. One branch undergoes deep feature extraction through *n* Bottleneck modules, followed by concatenation with the other branch and a channel-adjustment convolution. Subsequently, the SimAM module ingests this consolidated feature representation to execute an adaptive, weighted enhancement. This structural refinement substantially elevates the network’s representation selectivity, allowing it to highlight salient responses without introducing additional learnable parameters.

**Figure 5:**
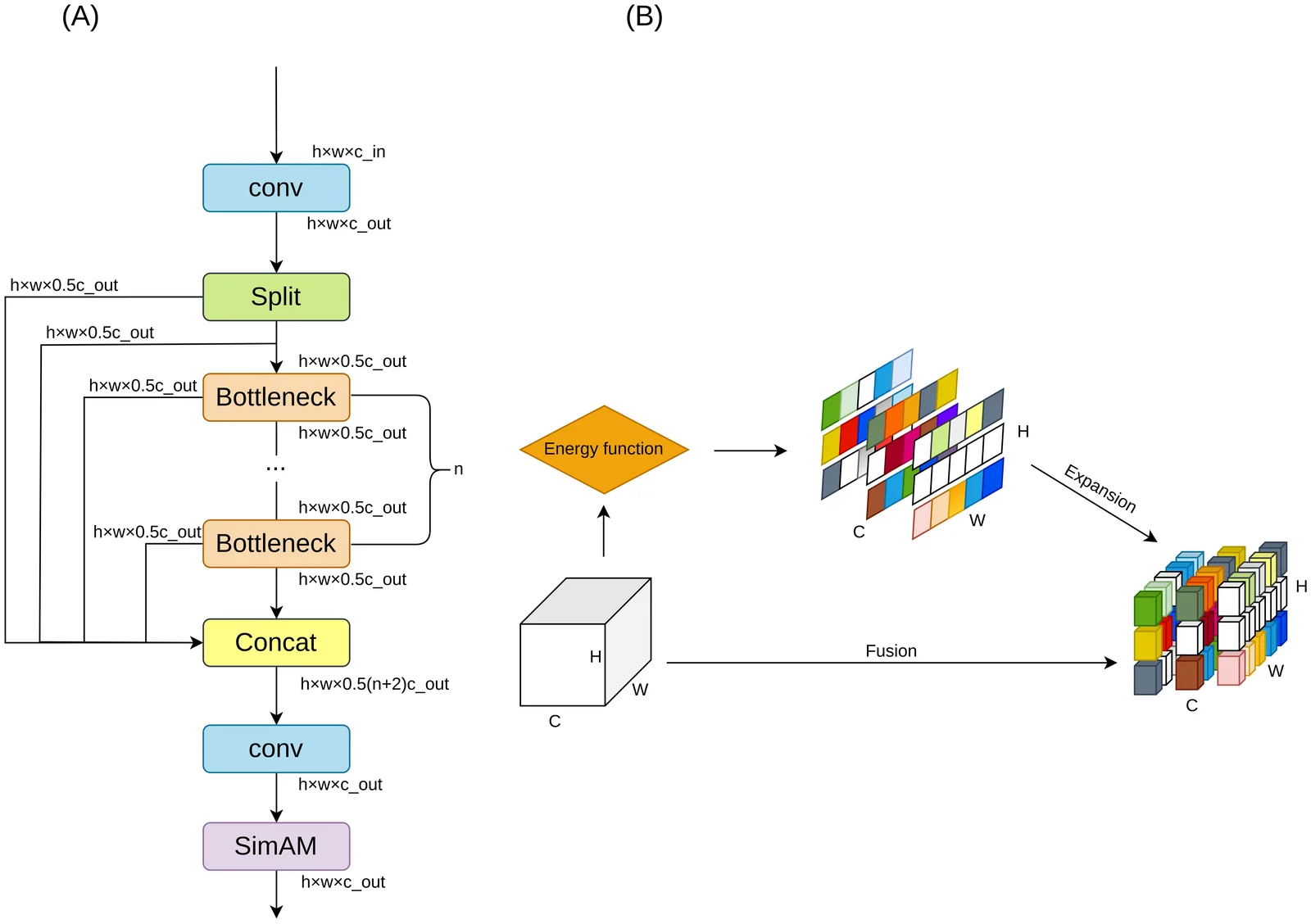
The total structure diagram of C2f_SimAM: **(A)** architectural design of C2f_SimAM, **(B)** schematic visualization of the core operations in SimAM.

Inspired by neuroscientific principles, SimAM evaluates 3D attention coefficients for individual units within the feature tensor via a rapid closed-form solution to an energy function. Without introducing extra parameters, this mechanism concurrently refines spatial and channel features to substantially boost the detection accuracy of dense, minuscule objects in complex backgrounds. Specifically, the perceptual importance of a target neuron *t* is e quantified by minimizing this tailored energy function, which is formulated as Equation 1:

(1) $$e_{t}\left(\omega_{t},b_{t},y,x_{i}\right)={\left(y_{t}-\hat{t}\right)}^{2}+\frac{1}{M-1}\sum_{i=1}^{M-1}{\left(y_{0}-{\hat{x}}_{i}\right)}^{2}$$

where *t* denotes the target neuron, *x_i_* represents other neurons, *ω_t_* and *b_t_* are the weight and bias, respectively, and *M=H×W* is the total number of neurons in the channel.

Minimizing this formulation essentially maximizes the ease with which the target neuron *t* can be linearly separated from the remaining units. For computational tractability, a regularization constraint is integrated alongside the assignment of binary labels (± 1), which yields the ultimate energy objective, as shown in Equation 2:

(2) $$e_{t}\left(\omega_{t},b_{t},y,x_{i}\right)=\frac{1}{M-1}\sum_{i=1}^{M-1}{\left(-1-\left(\omega_{t}x_{i}+b_{t}\right)\right)}^{2}+{\left(1-\left(\omega_{t}t+b_{t}\right)\right)}^{2}+\lambda \omega_{t}^{2}$$

The algebraic derivation of this optimization problem yields the definitive closed-form expression for the minimal energy status:

(3) $$e_{t}^{*}=\frac{4\left({\hat{\sigma }}^{2}+\lambda \right)}{{\left(t-\hat{\mu }\right)}^{2}+2{\hat{\sigma }}^{2}+2\lambda }$$

where $\hat{\mu }=\frac{1}{M}\sum_{i=1}^{M}x_{i}$ and ${\hat{\sigma }}^{2}=\frac{1}{M}\sum_{i=1}^{M}{\left(x_{i}-\hat{\mu }\right)}^{2}$ denote the spatial average and associated variance computed across the total neuronal units within the corresponding channel, respectively. This formulation demonstrates that a diminished value of $e_{t}^{*}$ denotes a more pronounced disparity between neuron *t* and its adjacent nodes, thereby signifying an elevated importance. Consequently, the attention weights for the neurons can be derived from $1/e_{t}^{*}$.

Guided by these principles, **Figure 5B** delineates the operational pipeline of the SimAM module. SimAM first calculates the mean and variance for each channel, then computes the minimum energy for each neuron according to Equation 3 to derive the attention weights. Ultimately, through element-wise multiplication, these generated weights are applied to the initial feature maps, thereby achieving an effective enhancement of critical features.

Compared with other attention modules, C2f_SimAM provides superior performance in both feature representation and computational efficiency. First, it significantly enhances the discriminative power of the model. By deriving 3D attention weights, SimAM identifies neurons that exhibit higher linear separability from their neighbors by simultaneously accounting for both spatial and channel dimensions. Specifically, neurons with greater disparity from their surroundings are assigned lower energy and, consequently, higher weights. This mechanism enables the module to autonomously amplify signals from lesion areas while effectively suppressing complex backgrounds and illumination noise. By integrating this refinement after the bottleneck layers, C2f_SimAM ensures that the feature maps transmitted to the detection head are well-calibrated, thereby mitigating information redundancy, while contributing to faster model convergence and superior overall detection accuracy. Second, this proposed module incurs no extra computational overhead while delivering such improvements. Unlike conventional attention mechanisms that require auxiliary convolutional or fully connected layers to learn weights, SimAM computes weights through an analytical solution derived directly from the current feature maps. This parameter-free property ensures that replacing standard C2f modules with C2f_SimAM maintains the original parameter count and FLOPs, providing a highly efficient approach to feature refinement suitable for real-time detection tasks.

##### 2.3.2.2 The histogram transformer block

Early-stage yellowing symptoms are characterized by localized small spots with faint features and sparse distribution. Furthermore, the complex environment presents significant challenges, as background elements (e.g., soil, weeds, and shadows) exhibit high grayscale similarity to the lesion areas. To enhance the model’s capability to extract minute yellowing features in complex field conditions and to mitigate interference from background factors, this study incorporates the Histogram Transformer Block (HTB) (Sun et al., 2025) as a feature enhancement module. This module sits at the tail end of the standard CIB (Compact Inverted Block), following all convolutional operations and preceding the residual connection. **Figure 6** delineates the global architecture of the proposed model.

**Figure 6:**
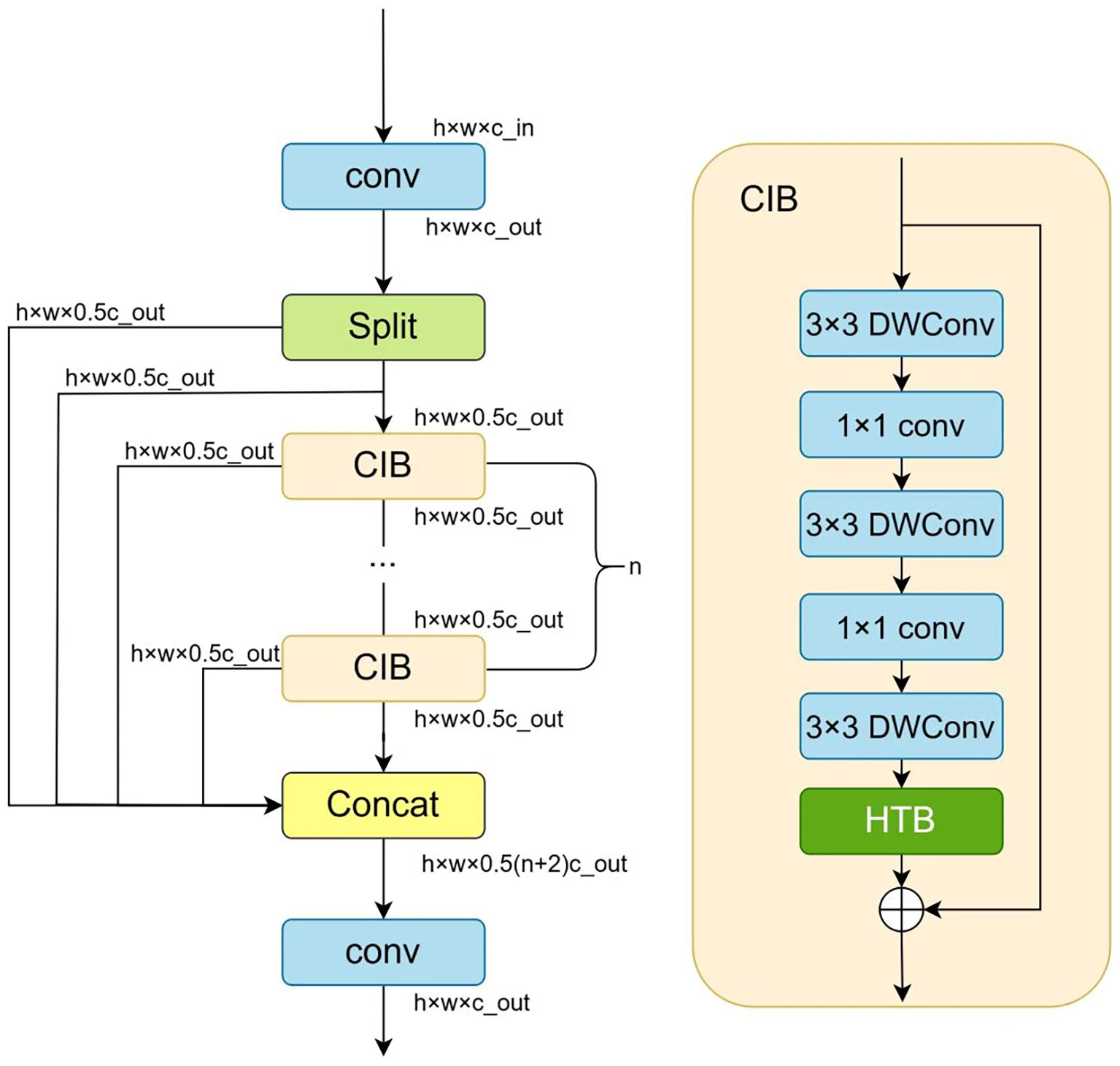
The structure diagram of C2fCIB_HTB.

Constituting the core architecture of the HTB module are the Dynamic-range Histogram Self-Attention (DHSA) mechanism and a complementary Dual-scale Gated Feed-Forward (DGFF) network. The entire architecture incorporates Layer Normalization (LN) paired with residual connections, thereby promoting a more stable training process. **Figure 7** delineates the step-by-step operational procedure. For a given input feature $F_{l-1}\in {\mathbb{R}}^{C\times H\times W}$ from layer *l-1*, the subsequent output *F_l_* of the HTB is formulated as Equations 4 and 5:

**Figure 7:**
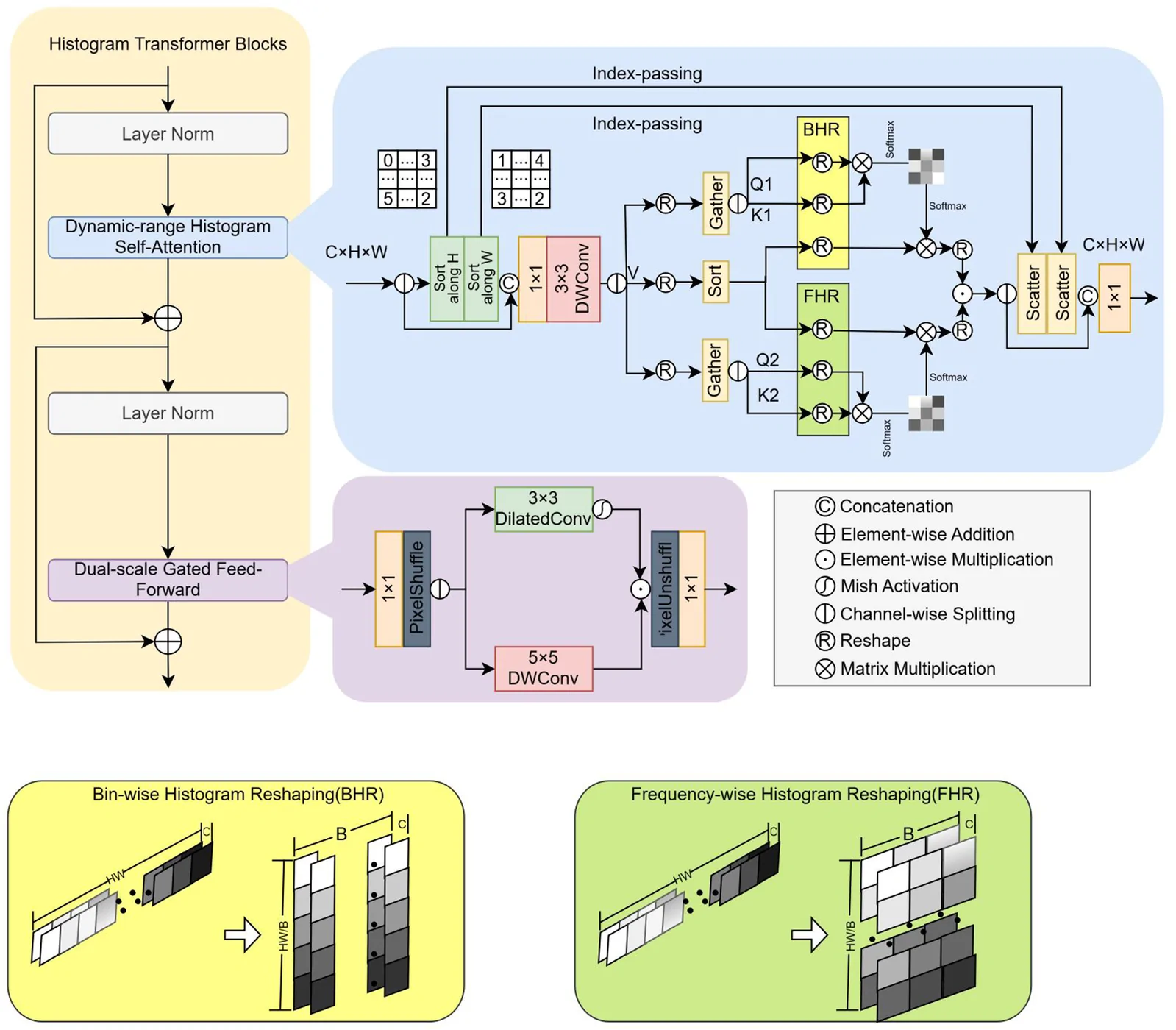
The structure diagram of HTB.

(4) $$F_{l}=F_{l-1}+DHSA\left(LN\left(F_{l-1}\right)\right)$$

(5) $$F_{l}=F_{l}+DGFF\left(LN\left(F_{l}\right)\right)$$

in which LN represents the Layer Normalization operation.

DHSA, serving as the architectural core of HTB, targets fine-grained details alongside comprehensive global contexts. Initially, features are rearranged via Dynamic-range Convolution. Specifically, a channel-wise partition divides the incoming features into twin streams, denoted as *F_1_* and *F_2_*. For the *F_1_* branch, dynamic sorting operations are executed in both horizontal and vertical directions. This process aggregates pixels with similar intensities into adjacent positions within the feature map, thereby forming regularized intensity patterns. Subsequently, the sorted *F_1_* is concatenated with the unsorted *F_2_*. Feature aggregation is then achieved as the features traverse a 1×1 convolution before a 3×3 depthwise separable convolution. Formally, this transition is mathematically defined as Equations 6 and 7:

(6) $$F_{1},F_{2}=Split\left(F\right),F_{1}=Sort_{v}\left(Sort_{h}\left(F_{1}\right)\right)$$

(7) $$F=Conv_{3\times 3}^{d}\left(Conv_{1\times 1}\left(Concat\left(F_{1},F_{2}\right)\right)\right)$$

Where *Split* denotes channel splitting; *Sort_h_* and *Sort_v_* represent horizontal and vertical sorting operations, respectively; the symbols $Conv_{3\times 3}^{d}$ and *Conv*_1×1_ designate the 3×3 depthwise and 1×1 pointwise convolution, with *Concat* specifying channel concatenation.

The features subjected to dynamic-range rearrangement are subsequently mapped into a value feature $V\in {\mathbb{R}}^{C\times H\times W}$ along with two query-key pairs $F_{QK,1},F_{QK,2}\in {\mathbb{R}}^{2C\times H\times W}$. The transformation process is thus given by Equations 8–10:

(8) $$V,d=Sort\left(R_{C\times H\times W}^{C\times HW}\left(V\right)\right)$$

(9) $$Q_{1},K_{1}=Split\left(Gather\left(R_{C\times H\times W}^{C\times HW}\left(F_{QK,1}\right),d\right)\right)$$

(10) $$Q_{2},K_{2}=Split\left(Gather\left(R_{C\times H\times W}^{C\times HW}\left(F_{QK,2}\right),d\right)\right)$$

Where $R_{C\times H\times W}^{C\times HW}$ denotes the reshaping of feature dimensions, while *Gather* isolates specified tensor values guided by the coordinate indices. Specifically, *d* represents the indices of the values obtained after the dynamic sorting process.

DHSA incorporates two parallel attention mechanisms: Bin-wise Histogram Reshaping (BHR), which facilitates holistic feature interaction across different bins, alongside Frequency-wise Histogram Reshaping (FHR), which performs fine-grained feature enhancement within each bin. Given the number of bins *B*, the self-attention mechanism operates according to Equations 11–13:

(11) $$A_{B}=Softmax\left(\frac{R_{B}\left(Q_{1}\right)R_{B}{\left(K_{1}\right)}^{\text{𝜏}}}{\sqrt{k}}\right)R_{B}\left(V\right)$$

(12) $$A_{F}=Softmax\left(\frac{R_{F}\left(Q_{2}\right)R_{F}{\left(K_{2}\right)}^{\text{𝜏}}}{\sqrt{k}}\right)R_{F}\left(V\right)$$

(13) $$A=A_{B}⊙A_{F}$$

Where *R_B_* and *R_F_* correspond to the reshaping operations of BHR and FHR, respectively; *k* denotes the attention head count; and ⊙ represents element-wise multiplication.

The features enhanced by DHSA subsequently enter the DGFF module to further fuse multi-scale information and strengthen feature representation. DGFF utilizes parallel convolutional kernels of varying scales (a large 5×5 kernel and a dilated 3×3 kernel in this study) to extract features. Through a gating mechanism, these multi-scale features are adaptively selected and fused. The exact architecture is given by Equations 14–16:

(14) $$F_{l,1},F_{l,2}=Split\left(Shuffle\left(Conv_{1\times 1}\left(F_{l}\right)\right)\right)$$

(15) $$F_{l,1}=Conv_{5\times 5}^{d}\left(F_{l,1}\right),F_{l,2}=Conv_{3\times 3}^{d,dilated}\left(F_{l,2}\right)$$

(16) $$F_{l+1}=Conv_{1\times 1}\left(Unshuffle\left(Mish\left(F_{l,2}\right)⊙F_{l,1}\right)\right)$$

Where *Shuffle* and *Unshuffle* denote pixel-rearrangement operations; $Conv_{5\times 5}^{d}$ represents a 5×5 depthwise convolution; $Conv_{3\times 3}^{d,dilated}$ refers to a 3×3 depthwise convolution with a dilation rate of 2; ⊙ and *Mish* correspond to the element-wise product and the activation function, respectively.

In summary, the HTB module effectively separates subtle yellow leaf lesion signals from complex backgrounds through intensity-guided pixel clustering, leveraging dynamic-range convolution and histogram partitioning. Subsequently, DHSA establishes global correlations among scattered lesions while refining local features through cross-bin and intra-bin interactions. Finally, DGFF is employed to further enhance feature representational capacity. Consequently, this architecture yields superior detection accuracy and resilience for identifying YLD within complex areca palm plantation environments.

##### 2.3.2.3 The GIoU loss

For object detection, localization accuracy is directly governed by the loss function used for bounding box regression. For bounding box regression, YOLOv10 uses Complete Intersection over Union (CIoU) (Zheng et al., 2022) by default. This loss accounts for three geometric aspects when comparing the predicted and ground-truth boxes: central point distance (normalized), overlap area, and aspect ratio. Its formulation is presented in Equations 17–19:

(17) $$V=\frac{4}{\pi^{2}}{\left(\tan^{-1}\frac{w^{gt}}{h^{gt}}-\tan^{-1}\frac{w}{h}\right)}^{2}$$

(18) $$\alpha =\{\begin{array}{c} 0,\;if\;IoU<0.5;\\ \frac{V}{\left(1-IoU\right)+V},\;if\;IoU\geq 0.5. \end{array}$$

(19) $$L_{CIoU}=1-IoU+\frac{\rho^{2}\left(b,b^{gt}\right)}{c^{2}}+\alpha V$$

Where $\rho^{2}\left(b,b^{gt}\right)$ is the squared Euclidean distance separating the centers of the predicted box and its ground-truth counterpart; *c* gives the diagonal length of the tightest enclosing box; *V* quantifies how consistent the aspect ratio is; and *α* functions as a balancing weight. CIoU adds an aspect ratio penalty term, which improves bounding box regression precision somewhat.

However, for the specific task of YLD detection, CIoU exhibits the following limitations. First, early-stage YLD lesions manifest as small chlorotic areas on leaves, accompanied by severe mutual occlusion among fronds. When the overlap between the predicted box and the ground truth box is minimal, the penalty weights assigned to center-point distance and aspect ratio in CIoU decrease significantly. As a result, the model struggles to obtain effective gradient feedback from the low-overlap predicted boxes during training. Second, the field environment is highly complex, containing background regions that share similar textures with the lesions. While the penalty terms in CIoU focus on the geometric attributes of the boxes, they are unable to explicitly handle localization offsets induced by background interference.

To tackle these problems, we replace the original CIoU with the Generalized Intersection over Union (GIoU) (Rezatofighi et al., 2019) loss function. The core concept of GIoU is to incorporate the smallest enclosing box as a penalty term based on the conventional IoU. The calculation formulas are presented in Equations 20, 21, and the relevant parameters are illustrated in **Figure 8A**.

**Figure 8:**
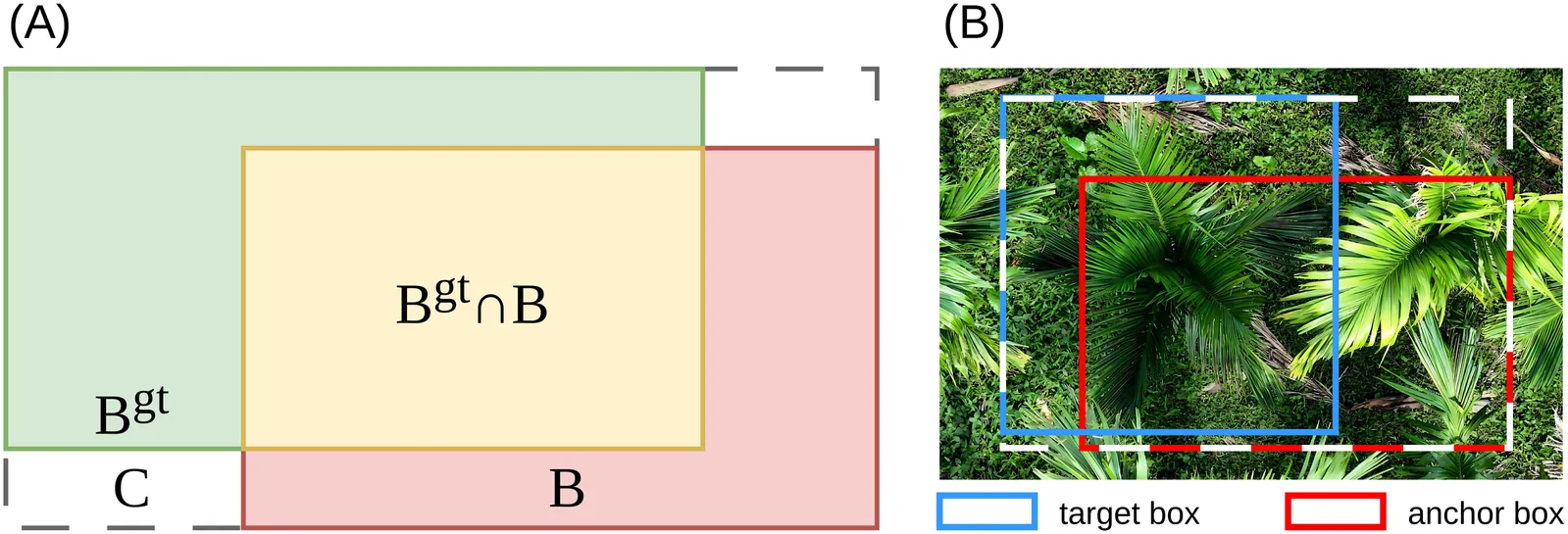
Principle of the GIoU loss function: **(A)** schematic, **(B)** training sample. *C* denotes the minimal enclosing box that covers both the real box and the predicted one.

(20) $$GIoU=IoU-\frac{\left|C-B^{gt}\cup B\right|}{\left|C\right|}$$

(21) $$L_{GIoU}=1-GIoU$$

Taking one training sample, **Figure 8B** shows how the target box (ground truth) and the predicted anchor box are positioned and overlap in image coordinates. This offers a clear view of the geometric parameters needed to compute the GIoU loss. Compared with CIoU, GIoU offers significant advantages in optimizing localization accuracy under complex conditions.

First, unlike conventional IoU, GIoU overcomes the lack of a usable gradient when the predicted and true boxes do not intersect (i.e., non-overlap cases). Because it includes the smallest enclosing box, GIoU keeps the loss function responsive to the spatial distance between the two boxes, even when they do not overlap. This provides a meaningful optimization trajectory for the model, which is particularly crucial for detecting early-stage lesions in scenarios characterized by dense occlusion. Second, GIoU evaluates alignment more thoroughly because it accounts for both the overlapping part and the non-overlapping region. In instances where areca palm leaves are mutually occluded, GIoU generates a continuous gradient signal even for slight offsets, guiding the predicted box to converge progressively toward the ground truth. Finally, GIoU retains scale invariance, facilitating the detection of lesions with varying sizes while maintaining low computational complexity, thereby ensuring that model training efficiency is not compromised.

During the inference and prediction phase, standard object detection networks typically employ traditional Non-Maximum Suppression (NMS) to eliminate redundant candidate bounding boxes. However, traditional NMS applies a hard-thresholding mechanism that directly sets the confidence scores of neighboring boxes to zero when their IoU with the highest-scoring box exceeds a predefined threshold. In densely occluded field environments, such a rigid deletion strategy inevitably leads to the erroneous suppression of genuine target boxes that are tightly clustered, thereby severely degrading the detection recall.

To overcome this structural limitation and optimize YOLOv10 for overlapping leaf scenarios, we seamlessly deploy a Soft-NMS-GIoU strategy specifically during the model’s prediction phase. Instead of applying a binary cutoff, Soft-NMS (Bodla et al., 2017) introduces a flexible attenuation function that continuously decays the confidence scores of alternative candidates based on their overlapping proximity. In this study, we utilize a Gaussian decay function for score adjustment, which is formulated as Equation 22:

(22) $$s_{i}=s_{i}\cdot e^{-\frac{GIoU{\left(M,b_{i}\right)}^{2}}{\sigma }}$$

Where *s_i_* denotes the updated confidence score of the candidate bounding box *b_i_* and *M* represents the bounding box with the maximum scoring confidence within the current iteration. Crucially, as highlighted in the formula, we replace the standard IoU metric with the Generalized Intersection over Union(GIoU) as the explicit core overlap criterion to achieve more robust spatial measurements. The hyper-parameter σ governs the severity of score suppression and is configured to its empirical optimal value of 0.5. Following score attenuation, a fine-grained filtering threshold of 0.001 is implemented to weed out non-target background predictions.

During implementation, this Soft-NMS-GIoU post-processing is executed iteratively: candidate boxes output by YOLOv10 are first sorted by confidence, and the scores of redundant neighbors are sequentially decayed based on their continuous GIoU distances to the highest-scoring anchor until the entire candidate pool is refined.

The integration of GIoU provides a scale-invariant and continuous spatial distance measure, while the Soft-NMS component prevents the premature elimination of closely clustered adjacent instances. Consequently, the Soft-NMS-GIoU strategy demonstrates enhanced robustness in severe leaf-occlusion environments, striking an ideal harmony between precision and recall.

## 3 Results and analysis

### 3.1 Experimental environment and parameter settings

To ensure the reliability and comparability of the experimental results, all experiments were conducted under identical hardware and software configurations. Although the raw UAV images were captured at 3,840 × 2,160 pixels, all images were uniformly resized and padded to 640 × 640 pixels as the fixed model input resolution for both training and inference. **Table 2** lists the detailed experimental environment configuration.

**Table 2:** The specifications of hardware and software.

Hardware and software	Configuration
CPU	16 vCPU Intel(R) Xeon(R) Gold 6430
GPU	NVIDIA GeForce RTX 4090
Optimizer	SGD
CUDA	11.8
System	Ubuntu 20.04.5
Environment configuration	Pytorch 2.0.0, Python 3.8.10
Epochs	100
Batch size	16
Input image size	640×640
Initial learning rate	0.01
Momentum	0.937
Attenuation	0.01

We assessed the proposed model using several metrics: precision, recall, mAP0.5, parameter count (Params), and FPS (frames per second) (Trigka and Dritsas, 2025).

### 3.2 Selection of object detection algorithms

#### 3.2.1 Comparative analysis of mainstream YOLO models

To identify the most suitable baseline model, five representative YOLO variants, YOLOv5s, YOLOv8s, YOLOv9s, YOLOv10s, and YOLO11s, are evaluated on a self-constructed YLD dataset. To guarantee fair comparisons, we trained and tested every model under the same experimental conditions. **Table 3** summarizes the results.

**Table 3:** Performance comparison among baseline models.

Model	Precision	Recall	mAP0.5	AP			FPS	Parameter/M
				Health	Mild	Severe		
YOLOv5s	0.789	0.823	0.856	0.865	0.796	0.935	79.932	**2.504**
YOLOv8s	0.790	0.812	0.868	0.881	0.785	0.939	**161.92**	3.006
YOLOv9s	0.863	**0.858**	0.904	0.912	0.855	**0.945**	79.362	7.168
YOLOv10s	**0.870**	0.840	**0.905**	**0.920**	**0.858**	0.937	108.321	8.037
YOLO11s	0.768	0.805	0.840	0.862	0.734	0.922	77.57	2.583

Bold values represent the best results according to the corresponding evaluation metrics.

From the perspective of detection accuracy, YOLOv9s and YOLOv10s demonstrate superior overall performance, achieving mAP0.5 values of 0.904 and 0.905, respectively, outperforming the other models. Notably, YOLOv9s attains the highest average precision (AP) of 0.945 in the severe category, indicating its strong capability in identifying advanced disease stages with prominent features. However, for the health and mild categories, YOLOv10s shows clear advantages. It achieves AP values of 0.920 for the health class and 0.858 for the mild class, both of which are the highest among all models. This finding is particularly important for early disease warning, as accurate identification of mild infections is critical for timely intervention and precision management.

In terms of computational efficiency, YOLOv8s achieves the highest inference speed, reaching 161.92 FPS. However, its detection accuracy is relatively limited, with only 0.868 mAP0.5 and a lower AP value of 0.785 for the mild category, which is significantly inferior to that of YOLOv10s. Although YOLOv9s achieves comparable accuracy to YOLOv10s, its inference speed is considerably lower, with an FPS of 79.362. In contrast, YOLOv10s strikes a favorable accuracy–efficiency trade-off, operating at 108.321 FPS. Compared with YOLOv9s, its speed is improved by 36.5% while maintaining the highest overall detection accuracy.

Overall, YOLOv10s demonstrates distinct advantages. It provides the best performance in the health and mild categories, which are critical yet difficult to distinguish, ensuring reliable early-stage disease detection; moreover, it strikes a favorable balance between detection precision and inference throughput, thereby satisfying the demands of both accurate detection and real-time operation.

#### 3.2.2 Attention mechanisms comparison

We integrated several representative attention modules into the YOLOv10s baseline for comparative analysis to evaluate their performance on YLD detection: CBAM, Coordinate Attention (CA) (Hou et al., 2021), Efficient Multi-Scale Attention (EMA) (Ouyang et al., 2023), Multi-Scale Context Attention (MCAttn) (Dai et al., 2026), Vision Transformer with Bi-Level Routing Attention (BiRAttn) (Zhu et al., 2023), SE, C2f_MCAttn (C2f-integrated MCAttn), and C2f_SimAM (C2f-integrated SimAM).

Specifically, CBAM, CA, EMA, MCAttn, and SE were inserted as independent attention modules into the feature extraction stages of both the backbone and neck, where they enhance intermediate representations through distinct channel-spatial recalibration strategies. BiRAttn was embedded into the C2fCIB blocks of the backbone, leveraging its bi-level routing attention to refine local feature aggregation. C2f_MCAttn and C2f_SimAM adopt a structural integration strategy, embedding MCAttn and SimAM directly within the C2f modules to enable joint optimization of feature extraction and attention weighting within unified computational units. Due to inherent differences in design philosophy, these methods are not strictly aligned in terms of exact insertion positions or implementation forms. To ensure a fair comparison, this study maintains consistent overall architecture, depth configuration, and training protocols across all experiments, while ensuring that all variants operate on the same levels of feature representations. Under these controlled conditions, the distinct feature modeling capabilities of each attention mechanism account for the performance differences observed. **Table 4** summarizes the comparative results.

**Table 4:** Comparison of attention mechanisms’ performance.

Model	Precision	Recall	mAP0.5	AP			FPS	Parameter/M
				Health	Mild	Severe		
w/o attention	0.870	0.840	0.905	0.920	0.858	0.937	108.321	8.037
CBAM	0.873	0.848	0.911	0.920	0.861	0.951	**122.346**	8.070
CA	0.867	0.858	0.912	0.921	0.861	0.953	111.050	8.063
EMA	0.872	0.852	0.913	0.923	0.864	0.952	103.739	8.040
MCAttn	**0.884**	0.846	0.916	0.922	0.870	**0.955**	107.281	8.563
BiRAttn	0.869	**0.878**	0.917	0.923	0.875	0.952	107.763	8.285
SE	0.871	0.860	0.918	0.929	0.874	0.950	110.930	8.038
C2f_MCAttn	0.870	0.871	0.920	0.935	0.874	0.950	101.325	8.289
C2f_SimAM	0.856	0.874	**0.921**	**0.939**	**0.877**	0.947	113.138	**7.826**

Bold values represent the best results according to the corresponding evaluation metrics.

As shown in **Table 4**, the C2f_SimAM model achieves the highest mAP0.5 at 0.921, representing a 1.6% improvement over the baseline (i.e., the original YOLOv10s without any attention). More importantly, it delivers the best results in the health and mild categories with AP values of 0.939 and 0.877, respectively. This improvement is particularly significant for early disease warning, as accurate identification of mild YLD is essential for effective precision management. Among the standalone attention modules, SE achieves the highest mAP0.5 (0.918) and health AP (0.929), while BiRAttn attains the highest recall (0.878) and mild AP (0.875). MCAttn performs well on the severe category (AP = 0.955) but shows modest gains on the mild category (AP = 0.870). CBAM and CA improve over the baseline yet remain suboptimal across most metrics. Notably, C2f_SimAM outperforms all standalone mechanisms, including those with higher computational costs such as BiRAttn and MCAttn.

Integrating attention mechanisms typically requires balancing detection accuracy against computational efficiency. MCAttn and C2f_MCAttn increase parameter counts to 8.563M and 8.289M (gains of 6.5% and 3.1% over the baseline, respectively), while their inference speeds drop to 107.281 and 101.325 FPS. BiRAttn and CA also introduce additional parameters (8.285M and 8.063M) without corresponding accuracy advantages over C2f_SimAM. In contrast, C2f_SimAM exhibits a distinct advantage by simultaneously improving both accuracy and efficiency. Its parameter count is reduced to 7.826 M, representing a 2.6% decrease relative to the baseline, while the inference speed increases to 113.138 FPS, with an improvement of 4.4%. This parameter-free design avoids the computational overhead of conventional attention structures, achieving synergistic enhancement of spatial and channel features without extra trainable weights.

**Figure 9** illustrates the feature activation distributions under different attention mechanisms. Notable differences exist among the methods in terms of feature focusing capability and background suppression. The baseline without attention generates dispersed responses, with high-response regions covering wide areas and exhibiting noticeable activation in non-target regions, indicating limited capability in selecting discriminative features and a susceptibility to background interference. CBAM, CA, EMA, and SE show enhanced responses in canopy regions but remain concentrated around the crown center with large activation areas, failing to focus on leaf-tip lesion zones; partial chlorotic regions remain unattended and background activation persists. MCAttn improves key-region responses, yet residual activation is still present in background areas, suggesting that its ability to suppress noise and enforce spatial constraints remains insufficient. BiRAttn activates most leaf-tip yellowing regions, but partial background areas still produce noticeable responses. C2f_MCAttn focuses primarily on leaf-tip chlorotic parts, though slight background activation and dispersion remain visible.

**Figure 9:**
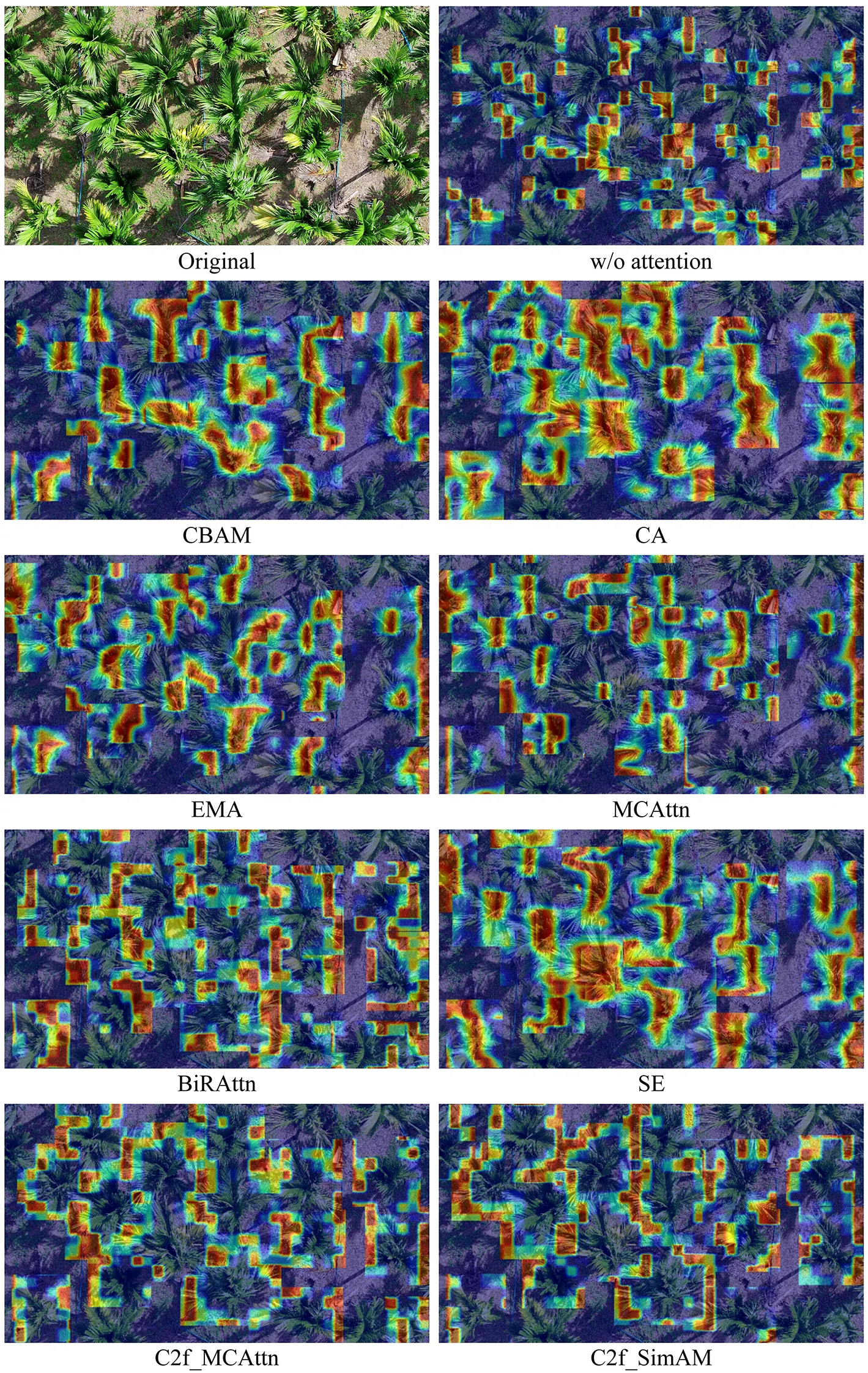
Attention mechanism feature map.

C2f_SimAM exhibits the most pronounced feature focusing effect, with attention responses concentrated on disease-bearing leaves, particularly at the leaf tips. This observation is consistent with the pathological progression of YLD, which typically initiates at the leaf tips and gradually spreads. This alignment indicates that the model effectively captures disease-relevant regions, thereby enhancing its ability to detect early-stage yellowing symptoms. No significant background activation is observed. These results confirm that the proposed C2f_SimAM mechanism effectively suppresses complex background interference while accurately localizing chlorotic leaf regions, offering the best trade-off between detection accuracy and computational efficiency.

#### 3.2.3 Comparison of loss functions

To evaluate how various loss objectives influence YLD detection efficacy, several representative loss functions, including SlideLoss (Zhang et al., 2025), Normalized Wasserstein Distance (NWD) (Wang et al., 2022), Adaptive Threshold Focal Loss (ATLF) (Yang et al., 2024), Distance Intersection over Union (DIoU) (Zheng et al., 2020), Minimum Point Distance Intersection over Union (MPDIoU) (Ma and Xu, 2023), Efficient Intersection over Union (EIoU) (Zhang et al., 2022b), Wise Intersection over Union v3 (WIoUv3) (Tong et al., 2023), and GIoU, were employed to replace the original CIoU loss in the YOLOv10s baseline model. **Table 5** summarizes the comparative results.

**Table 5:** Performance comparison among loss functions.

Model	Precision	Recall	mAP0.5	AP			FPS	Parameter/M
				Health	Mild	Severe		
CIoU	0.870	0.840	0.905	0.920	0.858	0.937	108.321	8.037
SlideLoss	**0.876**	0.858	0.914	0.926	0.873	0.944	116.683	8.037
NWD	0.867	0.872	0.915	0.928	0.873	0.944	107.057	8.037
ATLF	0.868	0.861	0.916	0.924	0.870	0.953	105.174	8.037
DIoU	0.866	**0.889**	0.917	0.929	**0.880**	0.944	116.262	8.037
MPDIoU	0.871	0.882	0.918	0.931	0.871	0.953	123.601	8.037
EIoU	0.873	0.874	0.920	**0.932**	**0.880**	0.947	117.039	8.037
WIoUv3	0.872	0.876	0.921	0.929	0.874	**0.958**	**129.193**	8.037
GIoU	0.865	0.881	**0.923**	**0.932**	**0.880**	0.957	120.257	8.037

Bold values represent the best results according to the corresponding evaluation metrics.

As shown in **Table 5**, GIoU loss yields the highest mAP0.5 at 0.923, outperforming CIoU by 1.8% and other loss functions by 0.2%–1.8%. This result indicates that GIoU provides stronger optimization capability for bounding box regression in the YLD detection task. Notably, GIoU achieves the highest average precision in the health and mild categories, with AP values of 0.932 and 0.880, respectively. Its severe AP reaches 0.957, marginally below WIoUv3 (0.958). Such consistently improved performance across categories is particularly important for YLD detection, where the simultaneous enhancement in the health and mild categories provides a reliable basis for early-stage disease screening.

Among the comparative methods, WIoUv3 achieves the highest inference speed at 129.193 FPS and the highest severe AP (0.958), yet its mAP0.5 (0.921) and mild AP (0.874) remain inferior to GIoU. EIoU attains comparable health AP (0.932) and mild AP (0.880) to GIoU, but its mAP0.5 (0.920) and severe AP (0.947) fall short. MPDIoU improves over CIoU with a mAP0.5 of 0.918 and a notable speed advantage (123.601 FPS), though its mild AP (0.871) is lower than that of GIoU. DIoU exhibits the highest recall (0.889) and competitive mild AP (0.880), yet its precision (0.866) and health AP (0.929) are suboptimal. SlideLoss improves precision (0.876) and speed (116.683 FPS) but shows limited gains in mAP0.5 (0.914). NWD and ATLF deliver moderate improvements in mAP0.5 (0.915 and 0.916, respectively) without achieving top performance in any single metric.

Beyond its superior accuracy, GIoU achieves an inference speed of 120.257 FPS, an improvement of 11.0% over the CIoU baseline and ranking second only to WIoUv3. This indicates that GIoU strikes an effective trade-off between spatial positioning precision and computational overhead. The incorporation of the smallest enclosing box as a geometric penalty enables GIoU to provide effective gradients even for non-overlapping predictions, a property that proves advantageous for mutually occluded areca palm leaves with blurred early-stage lesion boundaries. These results confirm that GIoU loss is well-suited for the YLD detection task, simultaneously enhancing localization accuracy and maintaining computational efficiency.

#### 3.2.4 Ablation study

To isolate the incremental performance gains attributable to each architectural modification, a series of ablation trials were executed. The proposed enhancements, encompassing C2f_SimAM, HTB, GIoU, and Soft-NMS-GIoU, were sequentially introduced into the baseline network. **Table 6** chronicles the detailed metric evolution across these configurations.

**Table 6:** Results of ablation experiments.

C2f_SimAM	HTB	GIoU	Precision	Recall	mAP0.5	AP			FPS	Parameter/M
						Health	Mild	Severe		
YOLOv10s			0.870	0.840	0.905	0.920	0.858	0.937	108.321	8.037
✓			0.856	0.874	0.921	0.939	0.877	0.947	113.138	**7.826**
	✓		0.882	0.859	0.911	0.924	0.872	0.938	74.588	8.933
		✓	0.865	**0.881**	0.923	0.932	0.880	0.957	**120.257**	8.037
✓	✓		0.871	0.867	0.922	0.933	0.875	0.956	70.724	9.098
✓	✓	✓	**0.887**	0.868	**0.929**	**0.944**	**0.883**	**0.960**	88.350	8.710

Bold values represent the best results according to the corresponding evaluation metrics.

As shown in **Table 6**, the YOLOv10s baseline achieves a mAP0.5 of 0.905, with Precision, Recall, and AP values of 0.870, 0.840, 0.920, 0.858, and 0.937 for Precision, Recall, Health, Mild, and Severe, respectively. Introducing C2f_SimAM increases mAP0.5 to 0.921 and Recall to 0.874, while the parameter count decreases from 8.037 M to 7.826 M and the inference speed increases from 108.321 to 113.138 FPS. The AP values for Health, Mild, and Severe also increase to 0.939, 0.877, and 0.947, respectively. These improvements indicate that C2f_SimAM enhances feature discrimination while introducing negligible computational overhead.

The individual HTB and GIoU experiments further reveal their complementary effects. The standalone HTB configuration improves Precision, Recall, and mAP0.5 to 0.882, 0.859, and 0.911, respectively. In particular, the Mild AP increases from 0.858 to 0.872, suggesting improved representation of weak disease symptoms. The HTB configuration also increases Health and Severe AP to 0.924 and 0.938, respectively. These improvements are consistent with the design of DHSA and DGFF, which enhance feature aggregation and multi-scale representation for small and weakly expressed YLD symptoms under complex canopy backgrounds. The corresponding inference speed decreases to 74.588 FPS because of the additional feature-processing operations. In contrast, replacing the original localization loss with GIoU produces a stronger improvement in overall detection performance, increasing Recall from 0.840 to 0.881 and mAP0.5 from 0.905 to 0.923, while also reaching 120.257 FPS. This indicates that GIoU primarily improves localization and regression performance without introducing additional parameters.

The interaction between C2f_SimAM and HTB is further demonstrated by their combined configuration. Compared with C2f_SimAM alone, adding HTB increases Precision from 0.856 to 0.871 and Severe AP from 0.947 to 0.956, although mAP0.5 changes only marginally from 0.921 to 0.922. This suggests that HTB provides complementary contextual and multi-scale information that is not fully captured by C2f_SimAM. The limited change in mAP0.5 therefore does not imply an absence of contribution, particularly when considering the class-wise AP and precision improvements.

With all three modifications integrated, the final model achieves the best overall performance, with Precision, Recall, and mAP0.5 of 0.887, 0.868, and 0.929, respectively. The AP values for Health, Mild, and Severe reach 0.944, 0.883, and 0.960, corresponding to improvements of 2.4, 2.5, and 2.3 percentage points over the baseline. Compared with the C2f_SimAM+HTB configuration, the addition of GIoU further increases mAP0.5 from 0.922 to 0.929 and improves the AP of Health, Mild, and Severe by 1.1, 0.8, and 0.4 percentage points, respectively. The final model also achieves 88.350 FPS with 8.710 M parameters. The ablation results demonstrate that C2f_SimAM, HTB, and GIoU provide complementary improvements: C2f_SimAM strengthens lesion-related feature discrimination, HTB enhances multi-scale contextual representation, and GIoU improves localization. Their integration consequently produces more consistent performance across the three YLD severity categories.

#### 3.2.5 Comparison with recent plant disease detection methods

To further evaluate the performance of the proposed model against recent domain-specific approaches, four YOLO-based plant disease detection methods, namely Cauli-Det (Uddin et al., 2024), TDD-YOLO (Chen et al., 2026), PYOLO (Wang et al., 2025), and DCH-YOLO11 (Cao et al., 2025), were selected for comparison. These methods were developed for cauliflower disease, tomato disease, plant disease, and citrus Huanglongbing detection, respectively, and incorporate task-oriented strategies including feature enhancement, attention mechanisms, and multi-scale feature fusion. The comparison was conducted on the same areca palm YLD test set under consistent evaluation criteria.

As shown in **Table 7**, the proposed model achieves the highest Precision (0.887) and mAP0.5 (0.929) among all compared methods. Its mAP0.5 exceeds those of Cauli-Det, TDD-YOLO, PYOLO, and DCH-YOLO11 by 2.5, 2.1, 1.7, and 1.0 percentage points, respectively. The proposed model also achieves the highest AP for the Health and Mild categories, with values of 0.944 and 0.883, respectively. The improved performance in these two categories is particularly relevant to YLD monitoring, as distinguishing healthy plants from mildly infected plants is essential for early disease assessment. DCH-YOLO11 achieves a slightly higher AP for the Severe category (0.965) than the proposed model (0.960), whereas the proposed model provides higher overall mAP0.5 and stronger performance in the Health and Mild categories. These results indicate that the proposed model provides more comprehensive detection performance across different YLD severity levels.

**Table 7:** Performance comparison with recent plant disease detection methods.

Model	Precision	Recall	mAP0.5	AP			FPS	Parameter/M
				Health	Mild	Severe		
Cauli-Det	0.838	**0.872**	0.904	0.919	0.846	0.947	108.535	11.313
TDD-YOLO	0.854	0.842	0.908	0.920	0.852	0.952	59.143	**2.579**
PYOLO	0.875	0.862	0.912	0.926	0.864	0.947	109.100	2.583
DCH-YOLO11	0.869	0.859	0.919	0.925	0.867	**0.965**	**130.101**	3.060
Ours	**0.887**	0.868	**0.929**	**0.944**	**0.883**	0.960	88.350	8.710

Bold values represent the best results according to the corresponding evaluation metrics.

Regarding inference efficiency, the proposed model achieves 88.350 FPS, which is sufficient for real-time or near-real-time UAV-based disease monitoring. Although some compared methods achieve higher inference speeds, their overall detection accuracy remains lower than that of the proposed model. The proposed model therefore provides a practical combination of high detection accuracy and efficient inference, supporting its application to large-scale areca palm plantation monitoring.

The comparative results demonstrate that the proposed model maintains its performance advantage not only over the YOLOv10s baseline but also over recently developed plant disease detection methods. In particular, its superior mAP0.5 and improved recognition of the Health and Mild categories highlight its effectiveness in fine-grained YLD severity detection under UAV imagery.

### 3.3 Model performance evaluation and misclassification analysis based on confusion matrices

To further analyze per-category classification performance, we constructed confusion matrices for the original model and our final model, as shown in **Figure 10**. The “background” entry in the confusion matrices does not represent an additional annotated class or a background-only negative sample. All images in the dataset contain areca palm plants, and only the three target categories (Health, Mild, and Severe) are annotated. In the detection confusion matrix, “background” represents unmatched detections or ground-truth targets: the background column corresponds to false-positive detections that cannot be matched to a ground-truth target, whereas the background row represents ground-truth targets that are not successfully detected.

**Figure 10:**
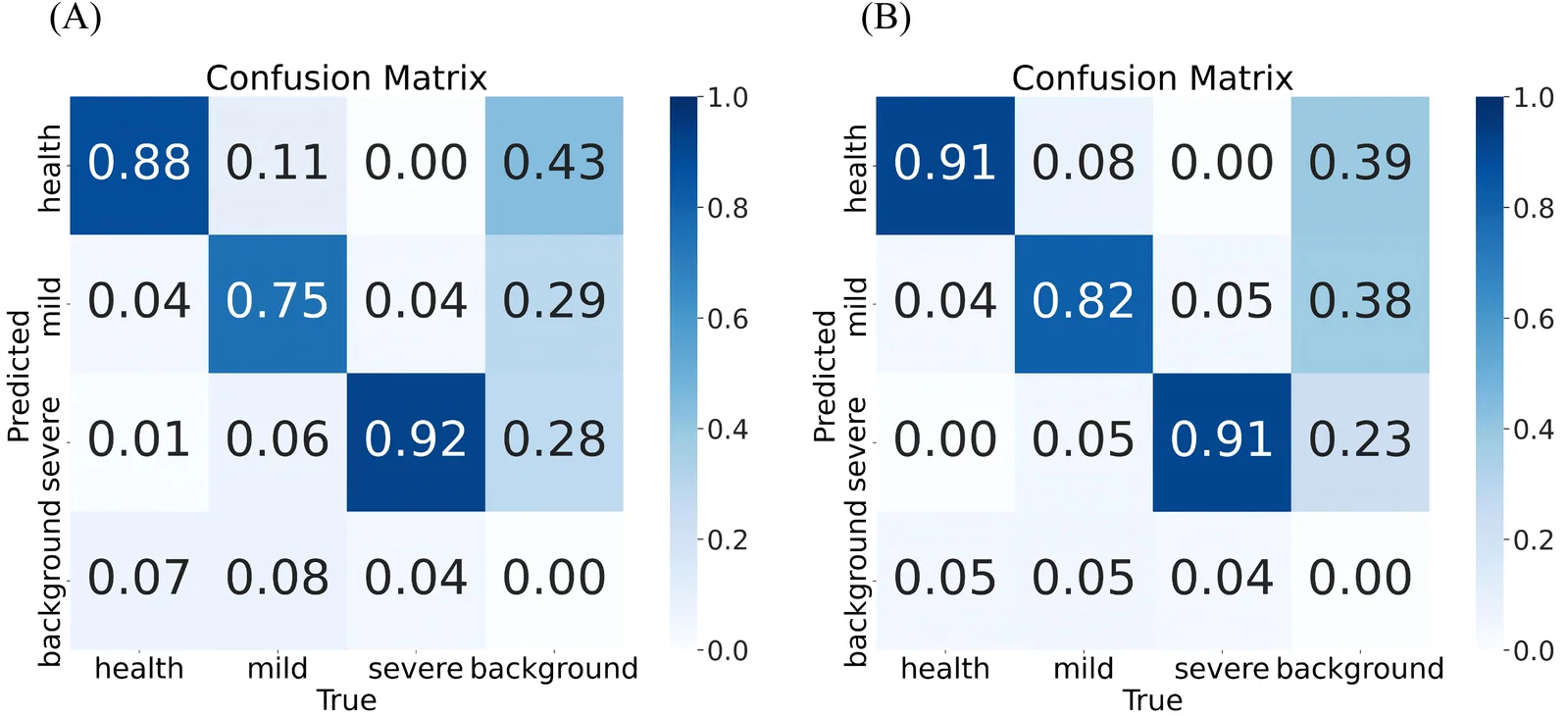
Confusion matrix: **(A)** YOLOv10s, **(B)** ours.

A comparison between **Figures 10A, B** shows that the improved model achieves higher values along the diagonal elements for all three disease severity levels, indicating enhanced classification accuracy. Specifically, the accuracy of the health category increases from 0.88 to 0.91, while the mild category shows a notable improvement from 0.75 to 0.82. The severe category maintains a high accuracy above 0.91. These results demonstrate an overall improvement in classification performance. The collective integration of the C2f_SimAM and HTB modules yields complementary feature enhancement, improving feature discrimination, particularly for the Health and Mild categories that share highly similar visual characteristics.

For both models, the primary misclassifications occur between adjacent disease severity levels, which is consistent with the progressive nature of disease development. In the baseline model, 11% of health samples are misclassified as mild, while 4% of mild samples are incorrectly predicted as health. In the improved model, these values are reduced to 8% and 4%, respectively, indicating a clear improvement in the fidelity of health classification. Moreover, the proportion of severe cases misclassified as background decreases from 0.28 to 0.23 in the improved model. This suggests that GIoU enhances localization accuracy in densely distributed and occluded scenarios.

### 3.4 Visualization analysis

To qualitatively appraise the model’s detection robustness, representative test cases featuring target occlusion, minute objects, and intricate background clutter were isolated for comparative visualization.

**Figure 11** presents a comparison of detection results under leaf occlusion conditions. The baseline model (YOLOv10s) exhibits noticeable missed detection and misclassification in such scenarios. Specifically, occluded healthy plants are not detected (as indicated by the white bounding boxes in **Figure 11A**), while certain healthy plants are incorrectly classified as mild (**Figure 11B**), and mild cases are misclassified as healthy (**Figure 11C**). Conversely, the proposed framework exploits the SimAM operator to sharpen representation concentration, while concurrently leveraging the GIoU loss formulation to augment bounding box precision. As a result, it not only mitigates detection failures induced by occlusion but also ensures precise differentiation between confounding health and mild states.

**Figure 11:**
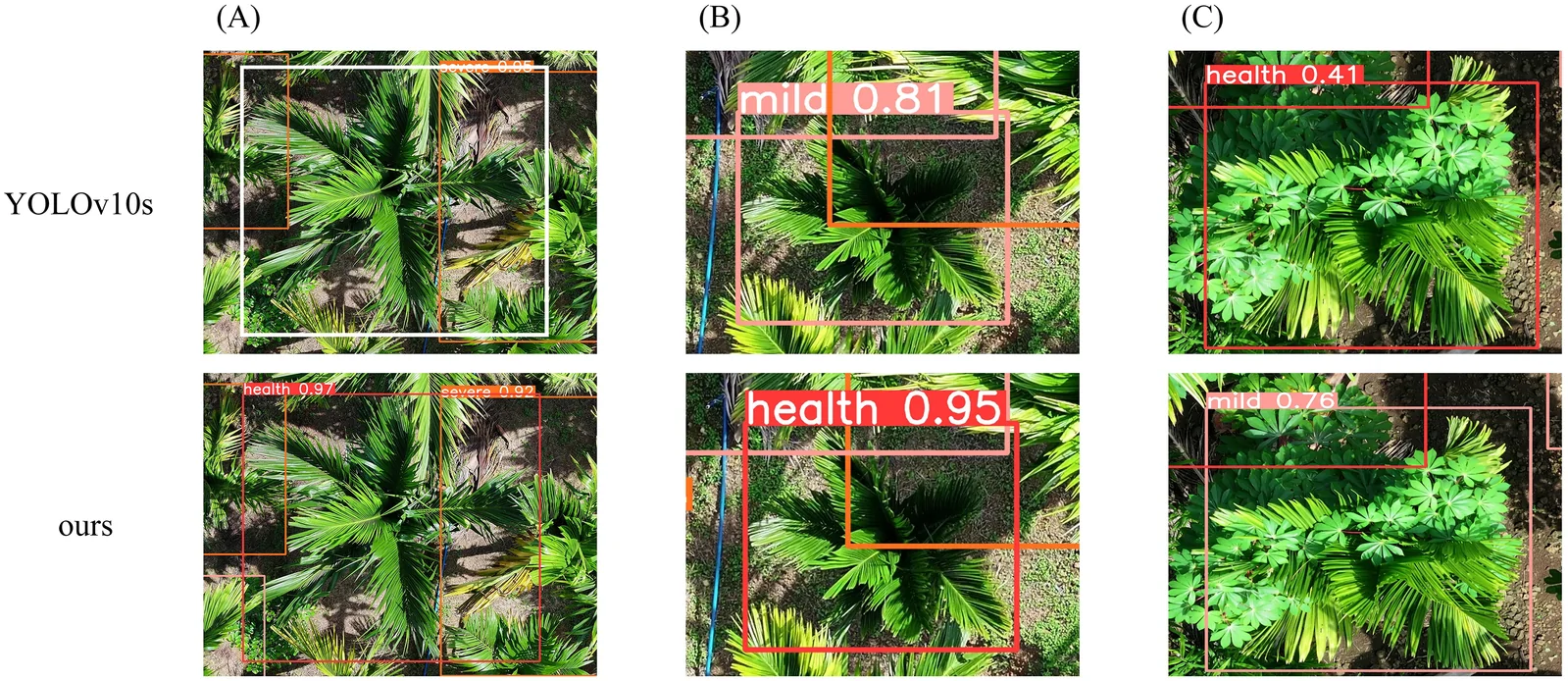
Optimization of occlusion problem: **(A)** missed detection, **(B)** and **(C)** false detection.

**Figure 12** presents a comparison of detection performance for small objects. Since the dataset is acquired from UAV-based remote sensing with a top-down perspective, some areca palm plants appear relatively small and occupy only a limited number of pixels in the images. The baseline network displays limited sensitivity to these small targets, culminating in a substantial rate of target omissions. This vulnerability is effectively resolved in the proposed architecture by integrating the HTB block, whose dynamic-range histogram self-attention mechanism vastly sharpens small-object representation. This approach enables better separation of small areca palm targets from the background, thereby significantly improving recall performance.

**Figure 12:**
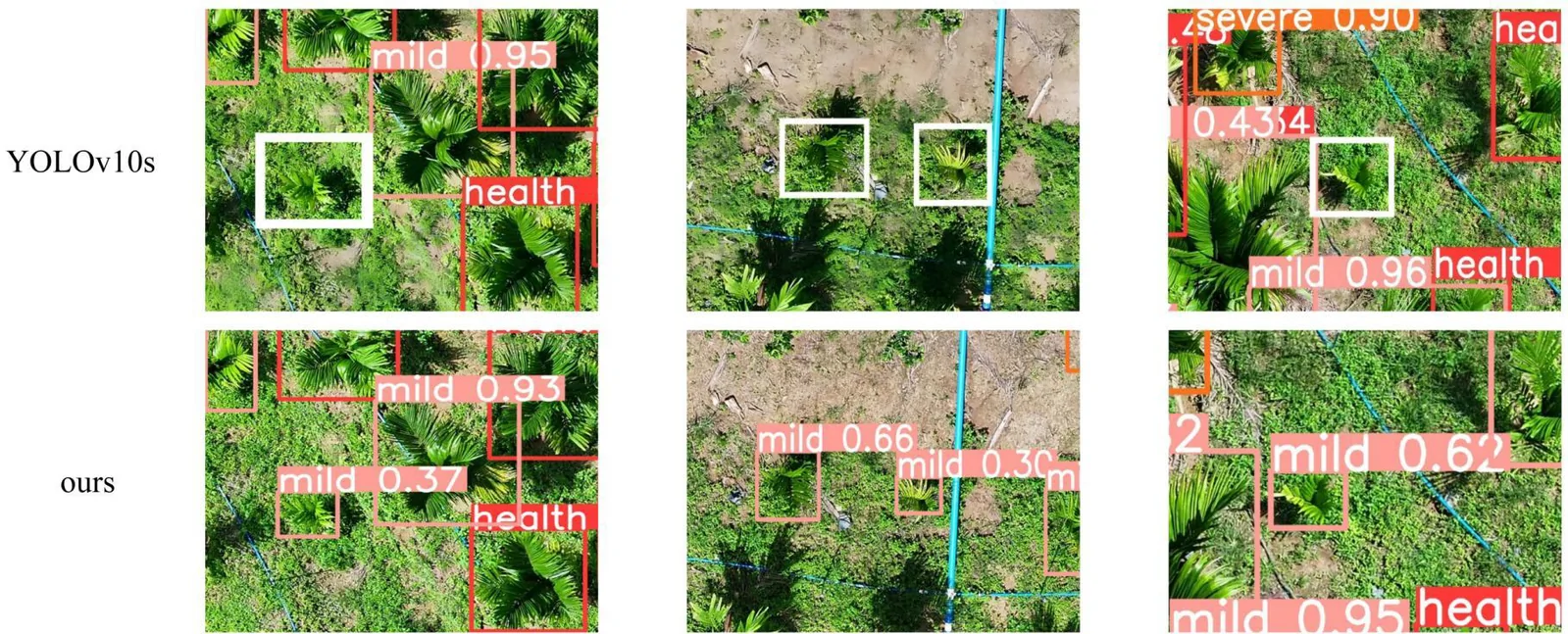
Optimization of small target missed detection.

**Figure 13** illustrates the detection performance under complex background conditions. In areca palm plantations, elements such as soil, weeds, and fallen leaves often exhibit grayscale characteristics similar to disease symptoms, which can easily lead to false detection. The improved model demonstrates a more concentrated attention distribution on yellowing regions of the leaves, effectively suppressing background interference. As a result, the occurrence of false positives is significantly reduced.

**Figure 13:**
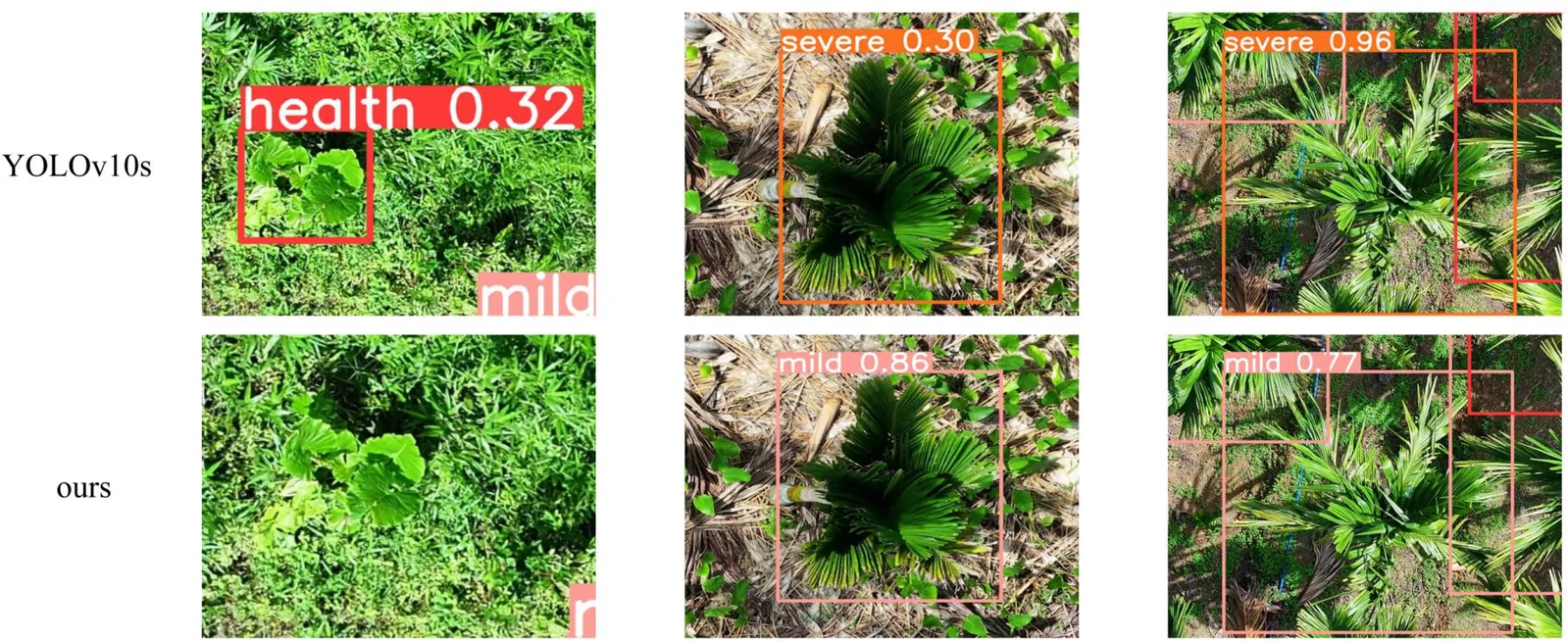
Optimization of background interference problem.

### 3.5 Field application results

The optimized model was deployed on UAV-acquired imagery from three real-world areca palm plantations to generate disease distribution maps, as illustrated in **Figure 14**. In these maps, healthy plants are marked in green, mildly infected plants in yellow, severely infected plants in red, and disease outbreak centers are indicated by purple circles, providing an intuitive visualization of the spatial clustering patterns of the disease.

**Figure 14:**
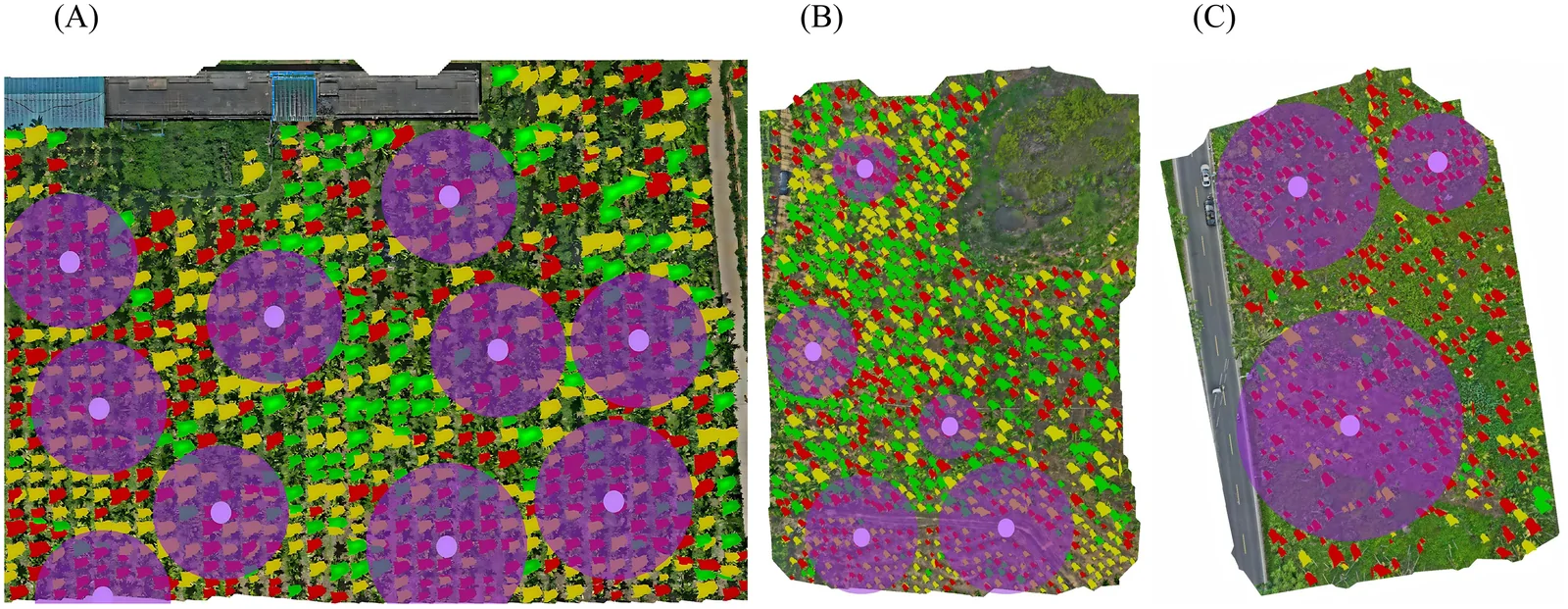
Spatial distribution map of YLD severity grades in areca palm plantations: **(A)** Plantation A, **(B)** Plantation B, **(C)** Plantation C.

The identification of outbreak centers is determined based on expert annotation experience and established plant protection survey protocols. First, a predefined spatial neighborhood is used as the analysis unit. If the proportion of detected yellowing plants (including both mild and severe cases) exceeds a threshold of 30% within the region, it is classified as a candidate outbreak area. Second, within these candidate regions, if the proportion of severely infected plants further exceeds 10% and exhibits a clear spatial clustering pattern, characterized by inter-plant distances significantly smaller than the average spacing within the plantation, the area is confirmed as an outbreak center.

Finally, connectivity analysis and spatial merging are applied to all detected outbreak regions to delineate complete outbreak zones, which are then visualized as circular regions. This approach effectively captures the true spatial distribution characteristics of the disease in plantation environments.

As shown in **Figure 14A**, a total of 10 outbreak centers are identified in Plantation A, distributed across the entire area with the highest density among the three sites. The sizes of these centers vary considerably, and some have merged into larger contiguous regions, forming extensive disease-affected zones. The large number and wide distribution of outbreak centers indicate that the disease has entered a widespread transmission stage, requiring urgent control measures.

In **Figure 14B**, five outbreak centers are detected in Plantation B, primarily located in the southern region. These centers exhibit clear clustering characteristics, with a high proportion of severely infected plants in the core areas. Moreover, a gradient distribution pattern is observed, with severe infection concentrated at the center, surrounded by mildly infected plants, and healthy plants at the periphery.

**Figure 14C** shows that three outbreak centers are identified in Plantation C, mainly distributed along the right side of the road. The left side of the plantation is occupied by a roadway, and the outbreak centers exhibit a distinct roadside distribution pattern, with the highest proportion of severe infections occurring in areas adjacent to the road.

**Table 8** presents the overall detection statistics for the three plantations, including the total number of areca palm plants, diseased plants, and disease rates obtained by the proposed model, together with the corresponding manual statistics. The model detected 2,269 plants and 1,761 diseased plants across the three plantations, corresponding to an overall disease rate of 77.61%, which is close to the manually calculated value of 79.91%. The results indicate that the proposed method can accurately estimate the overall disease distribution at the plantation scale, providing reliable support for large-scale field investigation and disease monitoring.

**Table 8:** Statistical summary of plantation disease detection results.

	Total number of areca palm plants	Total number of diseased plants	Disease rate
Plantation A (Model)	717	571	79.64%
Plantation B (Model)	1,104	760	68.84%
Plantation C (Model)	448	430	95.98%
Total (Model)	2,269	1,761	77.61%
Total (Manual)	2,364	1,889	79.91%

To further evaluate the model performance under unconstrained plantation environments, the detection results for the entire plantations were compared with manual statistics on a class-wise basis. The relative error was calculated as Equation 23:

(23) $$Relative\;error=\frac{\left|N_{Model}-N_{Manual}\right|}{N_{Manual}}\times 100\%$$

where *N_Model_* and *N_Manual_* represent the number of plants detected by the model and manually annotated, respectively. The class-wise validation results are presented in **Table 9**.

**Table 9:** Class-wise validation results on three plantation fields.

Plantation	Class	Model	Manual	Relative error
A	Health	146	135	8.14%
	Mild	258	290	11.03%
	Severe	313	328	4.57%
B	Health	344	326	5.52%
	Mild	388	434	10.60%
	Severe	372	385	3.38%
C	Health	18	14	28.57%
	Mild	87	95	8.42%
	Severe	343	357	3.92%

As shown in **Table 9**, the proposed model maintains good agreement with manual statistics across different plantations and disease severity categories. In Plantations A and B, the relative errors of all categories remain below 12%, indicating stable classification performance under different field conditions. The Severe category achieves the lowest relative error in all three plantations, suggesting that severe yellowing symptoms can be identified reliably despite variations in plantation background and disease distribution.

For Plantation C, the relative errors of the Mild and Severe categories are 8.42% and 3.92%, respectively. Although the Health category shows a relatively higher error, this plantation contains only a small number of healthy plants, making the relative error more sensitive to small numerical differences. Nevertheless, the model accurately reflects the overall class distribution and the dominant disease characteristics of the plantation.

These results demonstrate that the proposed model maintains stable class-wise detection performance under unconstrained plantation conditions, further validating its applicability for large-scale UAV-based monitoring of areca palm yellowing disease.

Overall, the model demonstrates robust generalization performance and practical applicability across the three representative plantation scenarios. The class-wise validation results further confirm the consistency between model predictions and manual statistics under unconstrained field conditions, while the generated disease distribution maps effectively reveal the spatial patterns of disease occurrence and accurately locate disease outbreak centers. These results provide strong evidence that the proposed method can be effectively deployed for large-scale plantation monitoring and precision disease management.

## 4 Discussion

### 4.1 Analysis of the effectiveness of model optimization strategies

Ablation experiments confirm the positive synergistic effects of the integrated modules on the robust YLD classification performance of the enhanced YOLOv10s architecture.

The empirical performance gain of the C2f_SimAM module originates from its parameter-free attention mechanism. Based on neuroscientific theory, SimAM evaluates the importance of neurons via an energy function and derives three-dimensional (3D) attention weights for each neuron in the feature map, thereby achieving synergistic enhancement of both spatial and channel features (Yang et al., 2021). This energy-based neuron evaluation, utilizing maximum-value pooling for feature map extraction, avoids the computational overhead of conventional attention structures (e.g., SE and CBAM). Consequently, it simultaneously increases mAP0.5 by 1.6%, reduces parameter count by 2.18%, and boosts inference speed by 4.45%. Heatmap visualizations (**Figure 9**) practically implicate that this mechanism effectively suppresses complex background interference (soil and weeds) to accurately localize chlorotic leaf regions.

For faint early-stage lesions exhibiting high grayscale similarity to backgrounds, the HTB module leverages a DHSA mechanism to aggregate intensity-similar pixels into histogram bins. This operation explicitly decouples foreground lesions from complex backgrounds (Sheng et al., 2025). Subsequent multi-scale feature fusion via DGFF substantially improves the extraction of minute chlorotic spots. While this spatial decoupling introduces computational overhead (reducing FPS to 70.724), the integration of the GIoU loss function offsets this bottleneck, restoring the FPS to 86.237 to maintain a favorable accuracy–efficiency trade-off.

The GIoU loss function directly mitigates localization bias under dense object occlusion. Unlike CIoU, GIoU incorporates the smallest enclosing box as a geometric penalty. This allows effective gradients to be provided even for non-overlapping predictions (i.e., where the predicted and ground-truth boxes share no area, IoU = 0) (Rezatofighi et al., 2019). This continuous gradient provision is particularly advantageous for mutually occluded areca palm leaves with blurred early-stage lesion boundaries. Empirically, GIoU reduces the misclassification rate of severe YLD regions as background from 0.28 to 0.23, significantly mitigating false negatives attributable to inaccurate bounding box regression.

The integrated model achieves 92.9% mAP0.5 (a 2.4% increase over the baseline), with precision and recall reaching 0.887 and 0.868, respectively. Confusion matrix analysis reveals a marked reduction in misclassifications between the visually similar “health” and “mild” categories, confirming the functional efficacy and diagnostic reliability of the proposed network mechanisms.

### 4.2 Challenges in detecting YLD and potential solutions

Although the improved model has demonstrated strong performance in the graded detection of YLD, it still faces two prominent challenges in practical applications: leaf specular reflection and interference from the natural yellowing of older leaves. These issues could be addressed through a synergistic approach that integrates polarization imaging techniques with data augmentation optimization strategies.

#### 4.2.1 Specular reflection from areca palm leaves

The surface of areca palm leaves is covered by a cuticle and a waxy layer, which can readily produce specular reflection under intense light. This phenomenon causes healthy leaves to appear yellowish or whitish while simultaneously deepening the apparent coloration of leaves with mild symptoms. As a result, a chain of misclassifications may occur, in which healthy leaves are misidentified as mild, and leaves with mild symptoms are misclassified as severe. A practical example of this issue is illustrated in **Figure 15**, where a healthy areca palm plant is misclassified as mild YLD due to specular reflection highlights, further underscoring the necessity of suppressing reflective interference. Research by Li et al. (2023) demonstrated that by constructing a polarized multispectral imaging system and developing the Polarization Fusion Specular Reflection Removal Index (PFSRRI), the correlation between spectral data and measured chlorophyll content (SPAD values) can be improved to *R*² = 0.955. Furthermore, the classification sensitivity for distinguishing healthy from stressed leaves reached 100%, substantially enhancing disease detection accuracy. These findings indicate that polarization imaging techniques can effectively differentiate specular reflection from diffuse reflection at the physical source, thereby fundamentally suppressing reflective interference. In future work, this technology could be incorporated to achieve precise severity grading of YLD.

**Figure 15:**
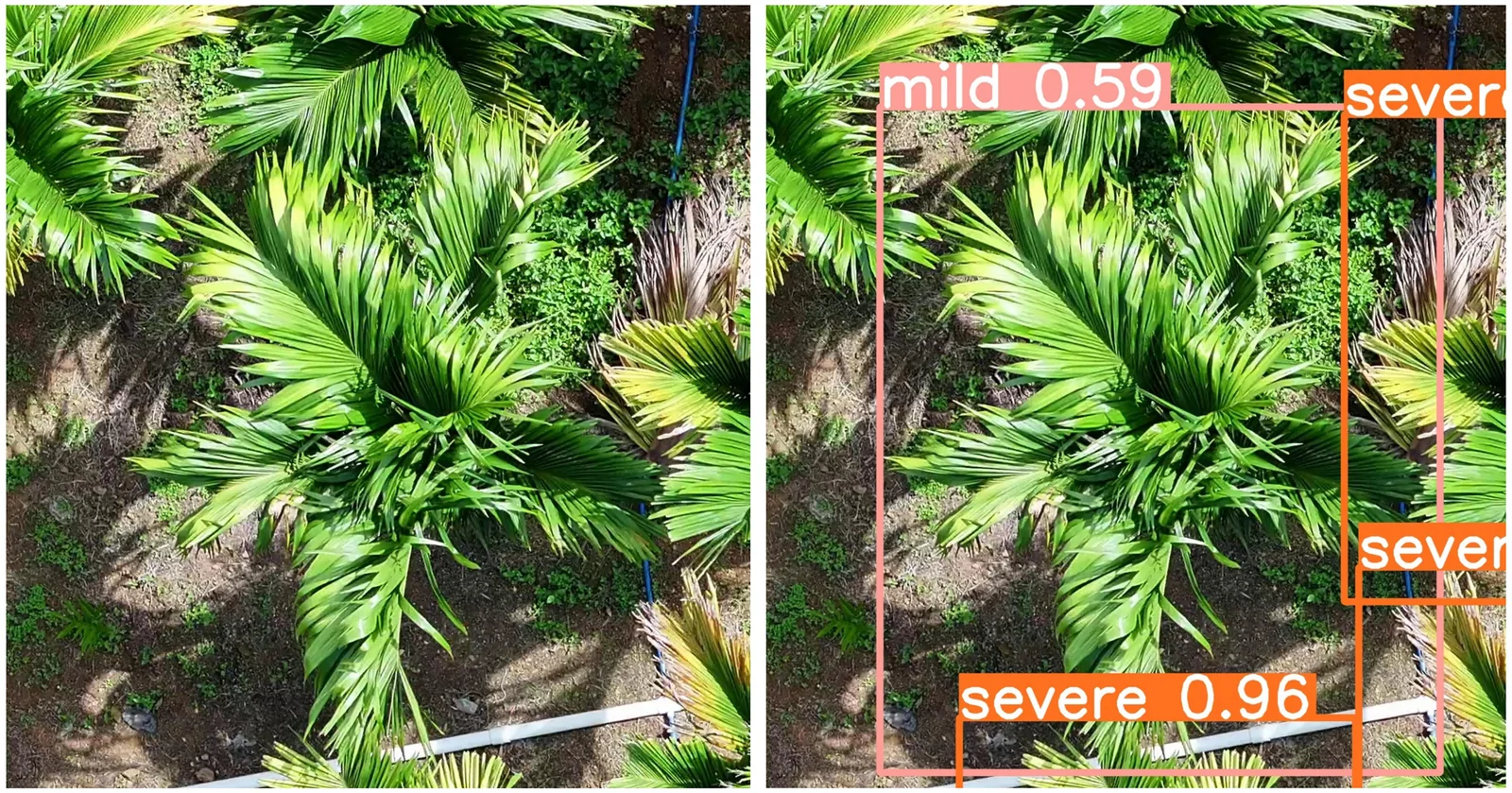
Specular reflection-induced YLD misclassification.

#### 4.2.2 Interference from natural yellowing of older areca palm leaves

During the natural growth process of areca palm plants, leaves in the lower canopy gradually undergo senescence, resulting in large areas of yellowed older foliage whose visual characteristics closely resemble those of disease-induced chlorosis. This physiological phenomenon significantly impairs the model’s ability to accurately discern the true disease status. This challenge is clearly illustrated in **Figure 16**, where a healthy areca palm plant bearing naturally senescent older leaves (**Figure 16A**, red box) is falsely detected as severe yellow leaf disease (**Figure 16B**), demonstrating the critical need to distinguish physiological yellowing from pathological symptoms. To address this issue, the dataset could be augmented with additional samples of plants bearing older leaves but free from disease, with these samples explicitly labeled as the “health” class. This enables the model to learn the subtle distinctions between naturally senescent leaves and pathologically yellowed ones. For instance, Madadum et al. (2025) tackled the class imbalance problem in watermelon leaf disease detection by proposing a data optimization method that combines the Segment Anything Model with photometric-geometric data augmentation. This approach increased the number of samples in the minority class from 235 to 1,023. Experimental results showed that YOLOv9t attained 0.92 mAP0.5 on the augmented dataset. This finding supports the use of such data augmentation for discriminating between physiological and pathological yellowing.

**Figure 16:**
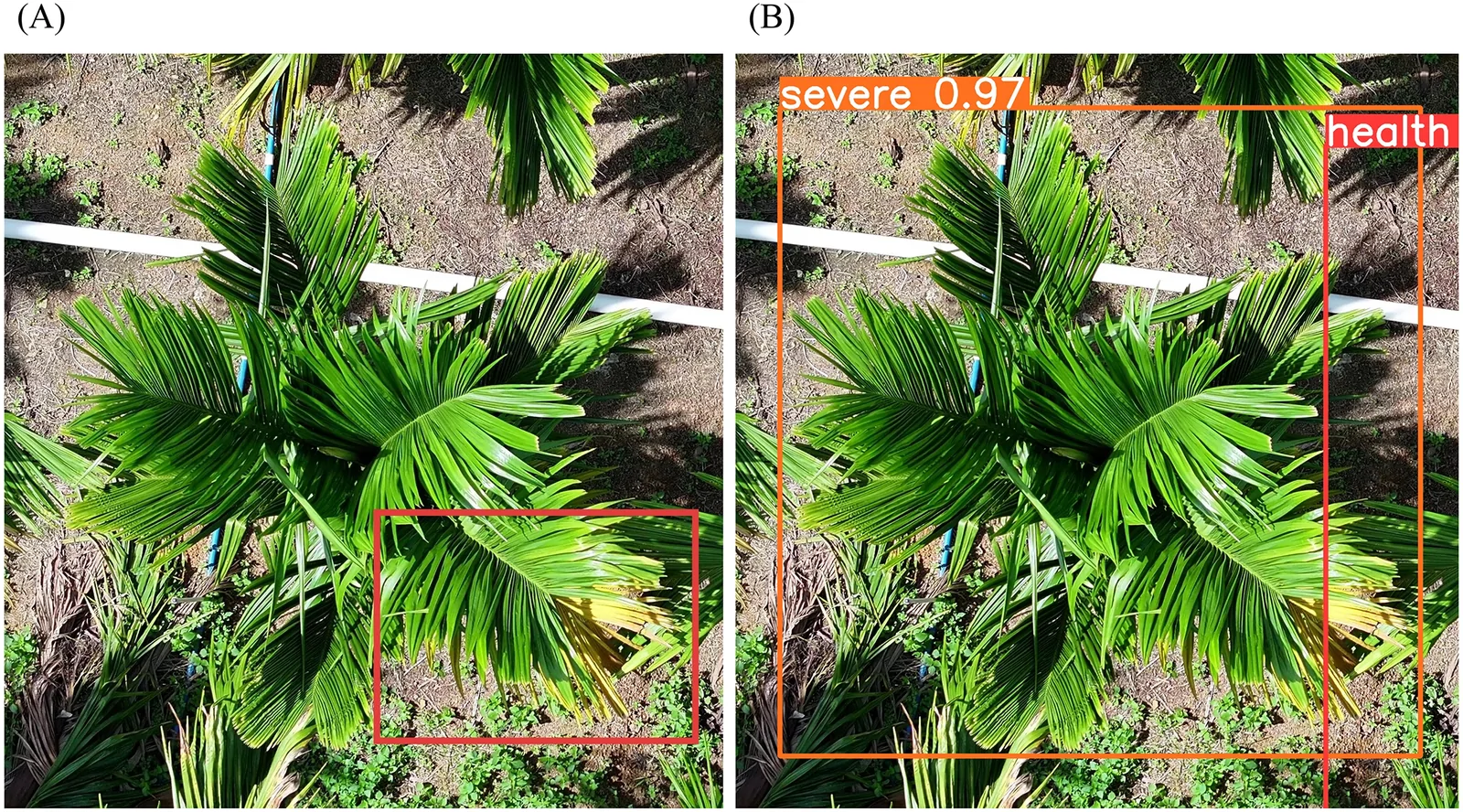
Misclassification of healthy areca palm caused by natural leaf senescence: **(A)** Healthy areca palm with senescent leaves (red box). **(B)** Falsely detected as severe YLD.

### 4.3 Integrated analysis of multimodal data and disease transmission mechanisms

Areca palm velarivirus 1 (APV1) can lead to YLD. The virus is transmitted mainly by mealybugs (*Ferrisia virgata* and *Pseudococcus cryptus*) in a semi-persistent, non-circulative manner (Zhang et al., 2022a). Concurrently, environmental factors such as temperature not only directly influence the population dynamics and migratory activity of the insect vectors but also modulate the expression of disease resistance in areca palm plants (Khan et al., 2022). Existing research indicates that remote sensing techniques can effectively capture spectral differences in plants induced by vector feeding damage, while the fusion of environmental sensor data with multispectral imagery can significantly enhance the accuracy of crop health assessment (Singh et al., 2016; Sajjad and Mumtaz, 2025). Given that single-dimensional information is insufficient to elucidate the complex mechanisms underlying disease onset and transmission, the establishment of a multimodal monitoring system that integrates disease phenotypes, vector insect distribution, and environmental factors holds considerable significance for enabling early warning and precise prevention and control.

This study achieved YLD detection using UAV-based RGB imagery and an optimized YOLOv10s model. The resulting spatial distribution map of YLD severity grades within the plantation provides an intuitive basis for the quantitative assessment of disease incidence and severity. Building on this foundation, the further integration of remotely sensed information on insect vectors with field-based environmental sensor data is expected to elucidate the underlying relationships between the spatial spread of YLD and driving factors such as mealybug population distribution, temperature, and humidity. Such integration would offer more comprehensive informational support for early warning of transmission risks and informed decision-making in the precise prevention and control of YLD.

## 5 Conclusion

This study addresses the challenges encountered in detecting areca palm yellow leaf disease (YLD) in UAV-based remote sensing imagery, including plant occlusion, difficulty in extracting features from small targets, interference from complex backgrounds, and subtle differences in visual characteristics across varying severity grades. A graded detection model for YLD based on an improved YOLOv10s architecture is proposed. Through theoretical analysis and experimental validation, the following conclusions are drawn:

Integrating the parameter-free SimAM attention into the C2f module (C2f_SimAM) sharpens the model’s focus on chlorotic lesions without extra parameters, suppressing complex background interference. The deep feature extraction stage incorporates the HTB module (C2fCIB_HTB), which uses DHSA and DGFF to enhance multi-scale lesion features. This design mitigates the challenge of detecting small targets—a difficulty arising from faint early-stage lesions that resemble background grayscale patterns. Replacing CIoU with GIoU overcomes gradient vanishing in low-overlap scenarios and bounding box regression bias under dense occlusion, and coupling it with Soft-NMS-GIoU further reduces false negatives and false positives. The final model attains 92.9% mAP0.5 (+2.4 pp over YOLOv10s), precision of 88.7%, recall of 86.8%, and an inference speed of 88.350 FPS. The misclassification rate between “health” and “mild” categories drops markedly, with recall for the two classes rising to 91% and 82%, respectively, demonstrating improved accuracy and robustness for YLD severity grading.

The optimized model was applied to an actual areca palm plantation, generating a visual disease distribution map and successfully identifying the outbreak epicenter. This field application confirms that the model is both practical and reliable under real production conditions. It thus offers a solid technical basis for large-scale surveillance and targeted management of YLD.

## Data Availability

The datasets generated during the current study are available from the corresponding author upon reasonable request.
